# Supplementary material for: Sodium-Doped 3-Amino-4-hydroxybenzoic Acid: Rediscovered Matrix for Direct MALDI Glycotyping of O-Linked Glycopeptides and Intact Mucins
Source: Int J Mol Sci. 2023 Nov 28;24(23):16836. doi: 10.3390/ijms242316836 (PMC10706304; doi:10.3390/ijms242316836)
Supplement: Supplementary file 1 [file ijms-24-16836-s001.zip › ijms-2720067-supplementary.pdf]

Supplementary Materials:

Sodium-Doped 3-Amino-4-Hydroxybenzoic Acid: Rediscovered Matrix for Direct MALDI Glycotyping of *O*-linked Glycopeptides and Intact Mucins

Shogo Urakami[1], Hiroshi Hinou[2]*

1 Graduate School of Life Science, Hokkaido University, N21W11, Kita-Ku, Sapporo 001-0021, Japan.

2 Frontier Research Center for Advanced Material and Life Science, Faculty of Advanced Life Science, Hokkaido University, N21W11, Kita-Ku, Sapporo, 001-0021, Japan.

*Corresponding author. Email: hinou@sci.hokudai.ac.jp.

TABLE OF CONTENTS

Supplementary Figures S1~S29

Supplementary Tables S1~S22

SUPPLEMENTARY FIGURES

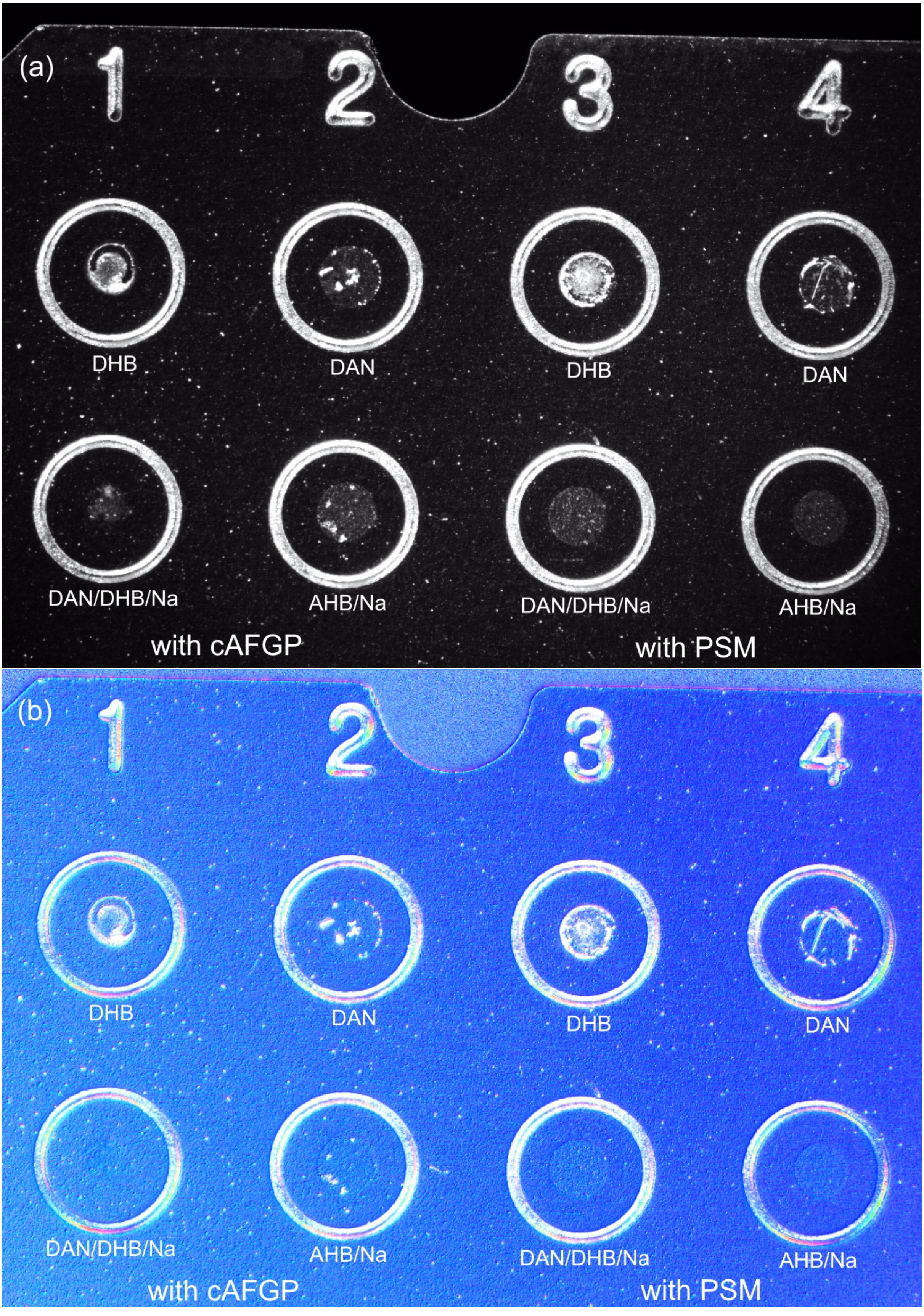

Figure S1. Morphological snapshot of the matrices used in this study after drying with samples on μFocus-type MALDI target plate taken by polarization camera: (a) with intensity mode, (b) with angle of linear polarization mode.

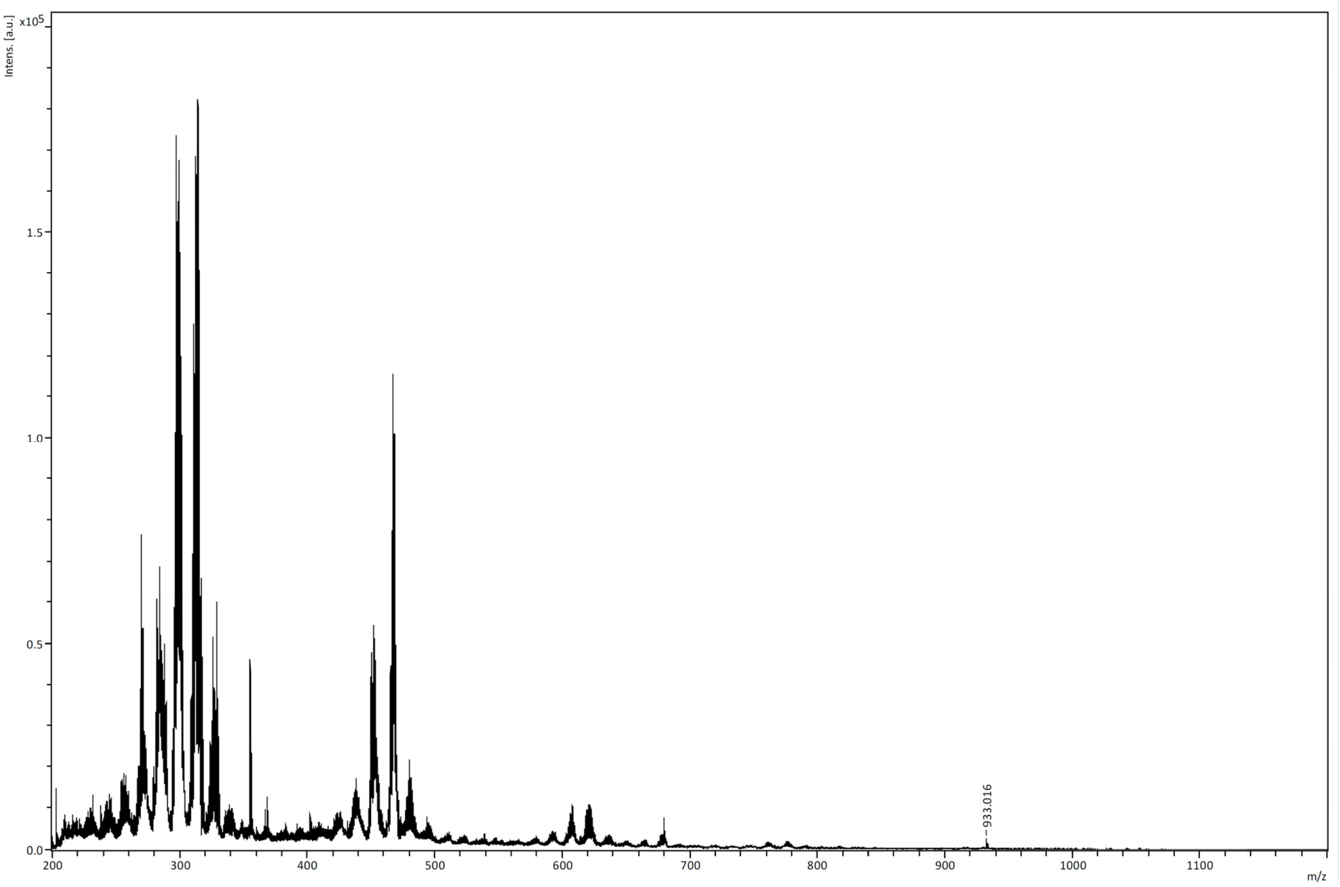

Figure S2. MALDI-ISD mass spectrum of *O*-glycans in PSM (1 μgμL^{-1}) with DAN matrix.

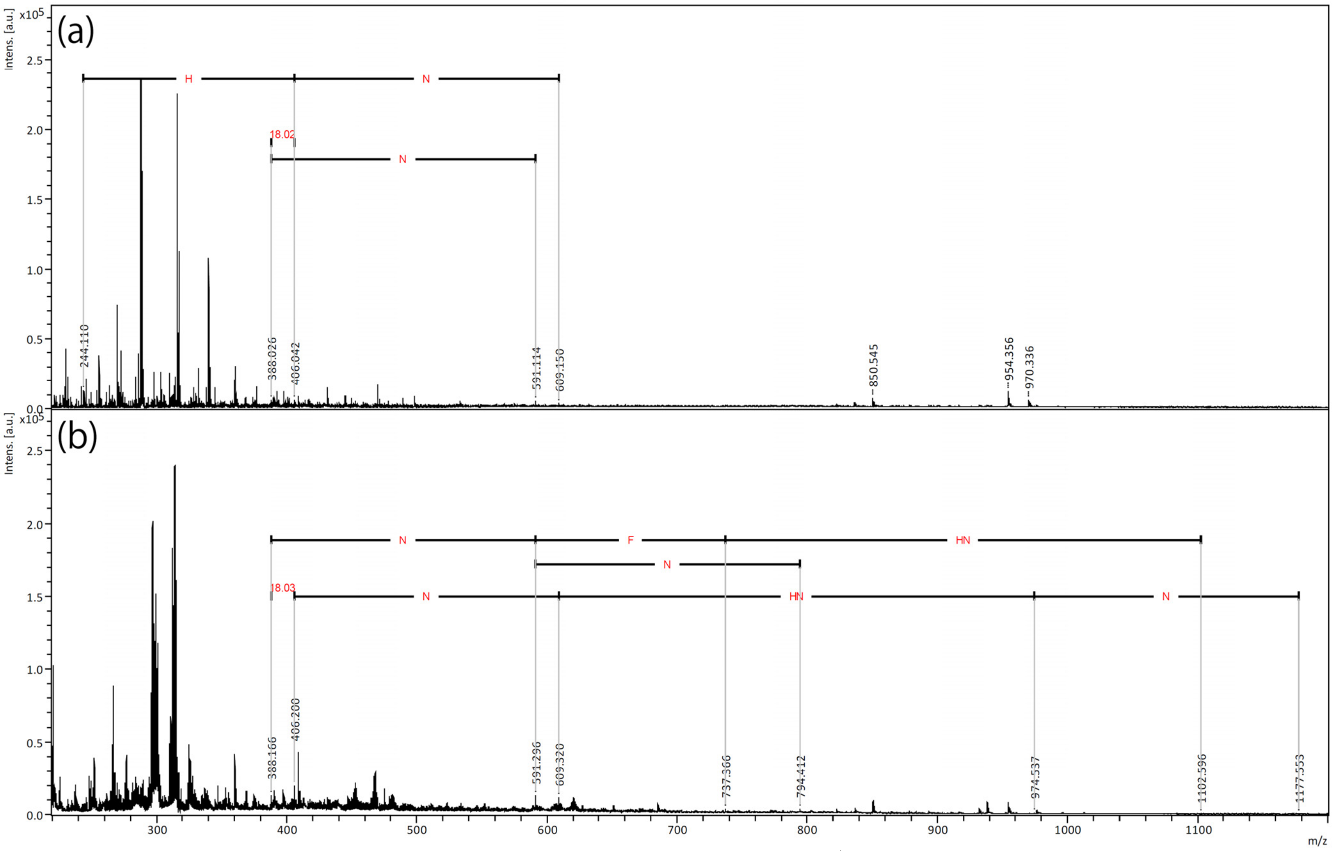

Figure S3. MALDI-ISD mass spectrum of *O*-glycans in PSM (1 μgμL^{-1}): (a)with DHB, (b) with DAN/DHB/Na.

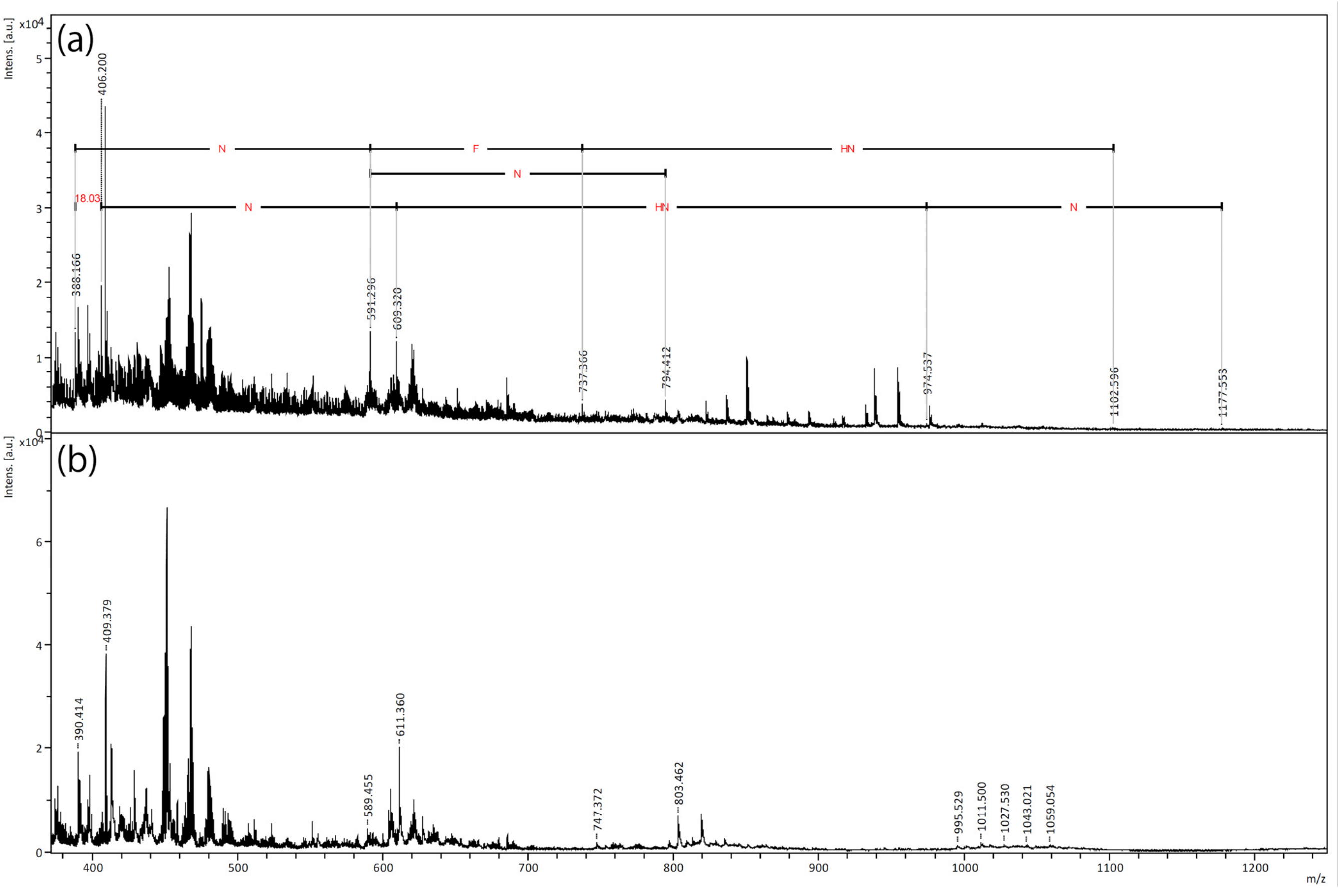

Figure S4. MALDI mass spectrum of DAN/DHB/Na matrix: (a) with PSM, (b) without sample.

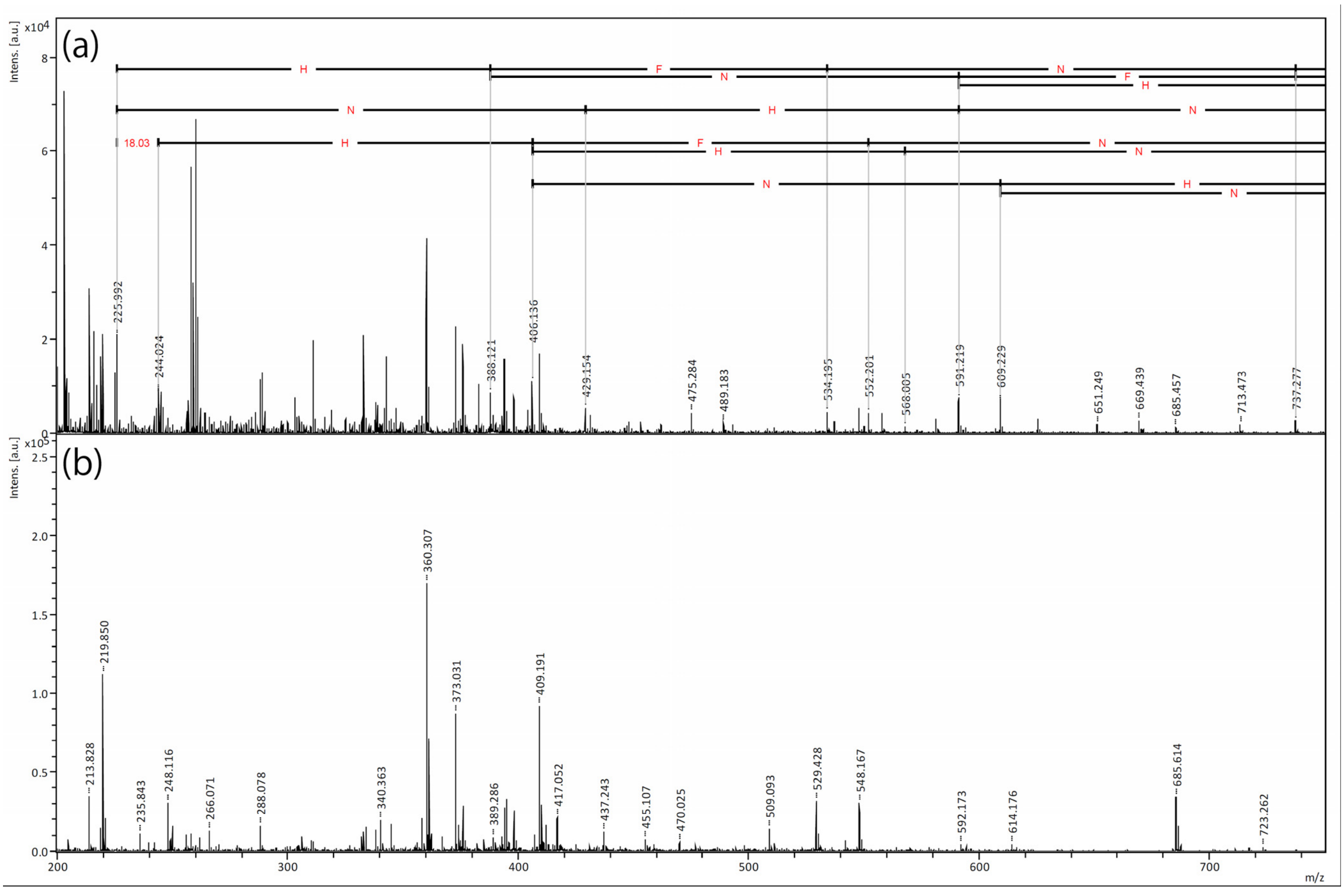

Figure S5. MALDI mass spectrum of AHB/Na matrix from *m/z* 200 to *m/z* 750: (a) with PSM (b) without sample.

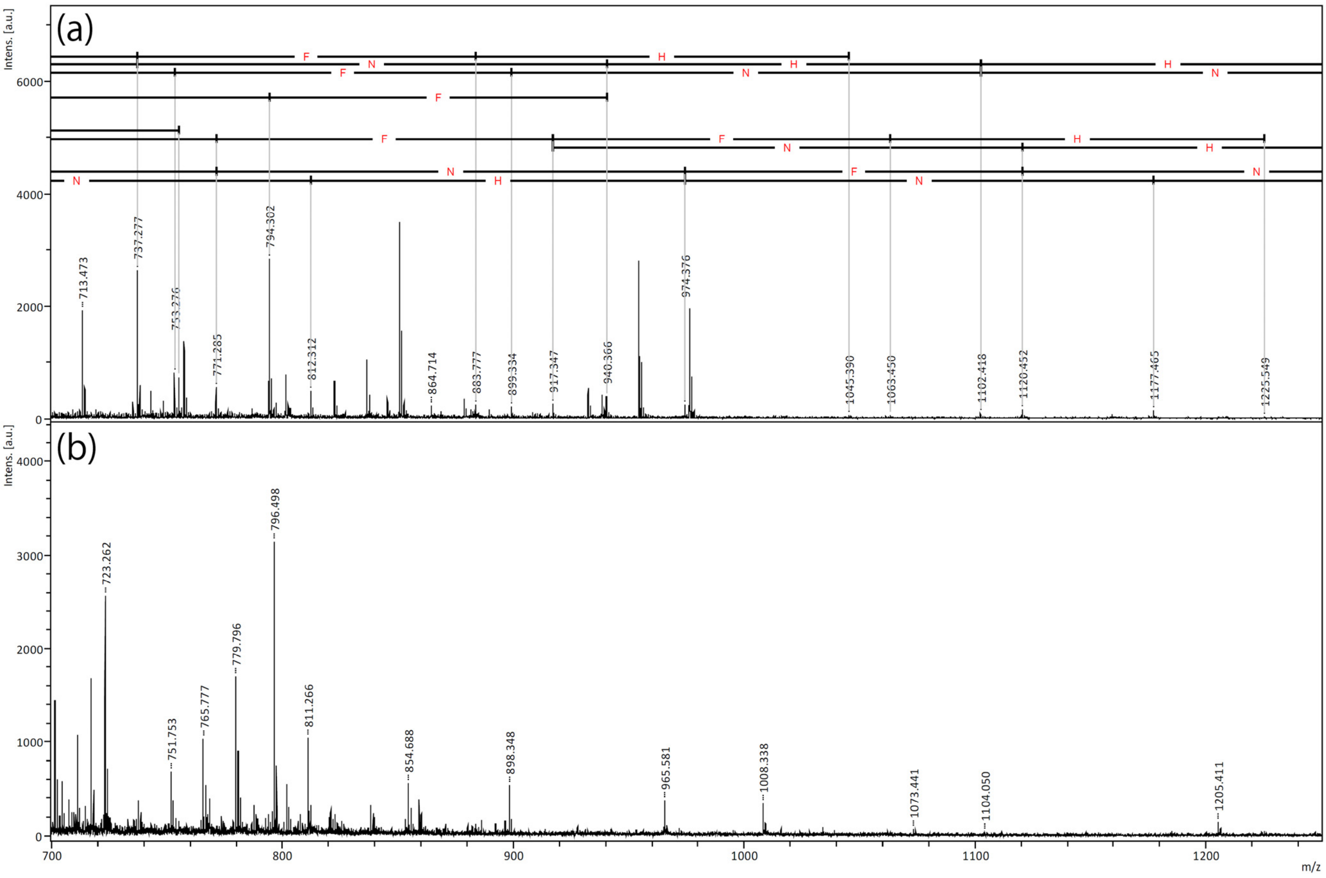

Figure S6. MALDI mass spectrum of AHB/Na matrix from *m/z* 700 to *m/z* 1250: (a) with PSM (b) without sample.

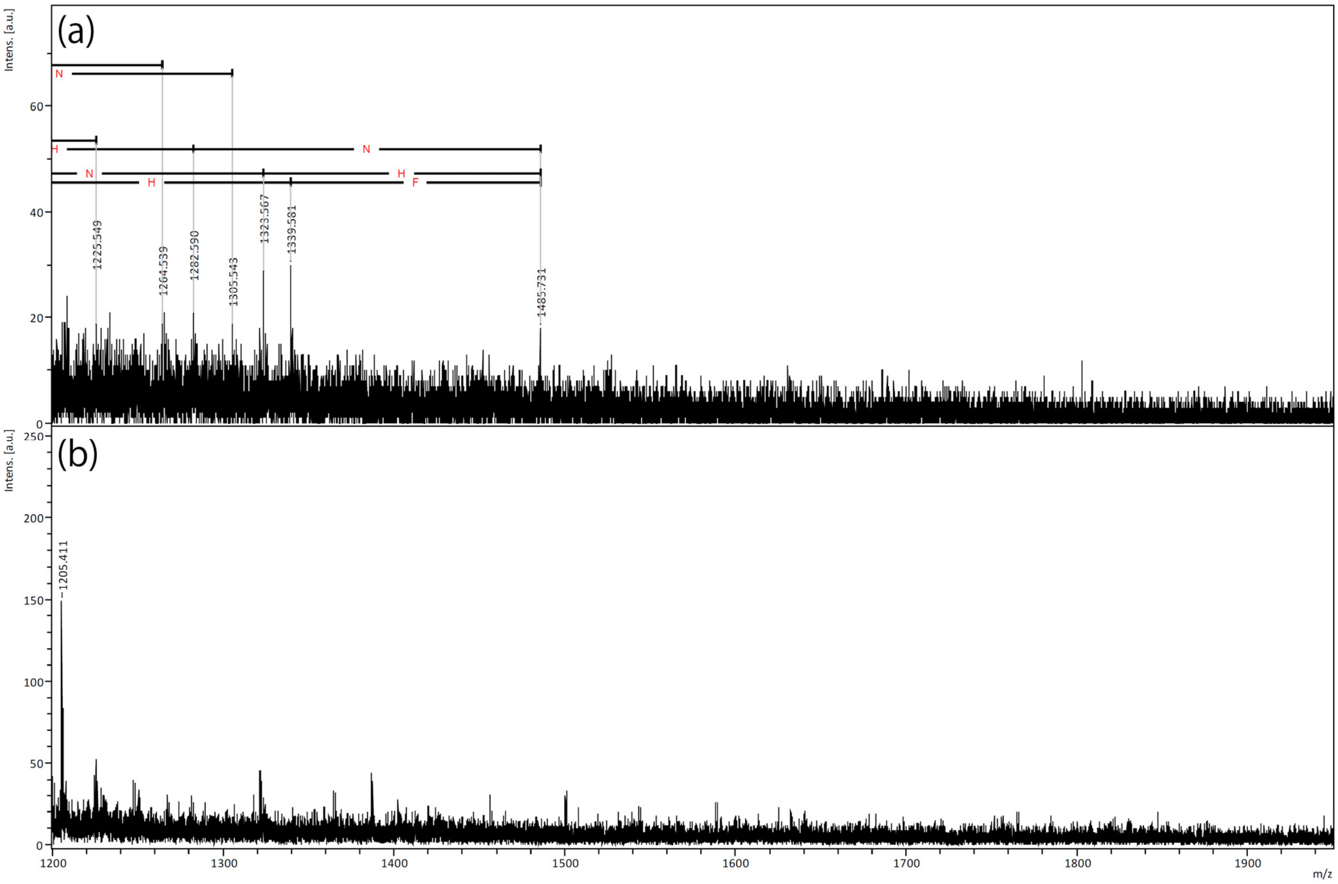

Figure S7. MALDI mass spectrum of AHB/Na matrix from *m/z* 1200 to *m/z* 1750: (a) with PSM (b) without sample.

Figure S8~S13. Δ*m/z* 123 comparisons of MALDI mass spectrum of PSM: (a) with AHB/Na, (b) with BOA/DHB/Na. (If the BOA-tagged glycan structure was detected as B ion, the *m/z* difference would be 123. Therefore, the *m/z* range was shifted by 123 for comparison.)

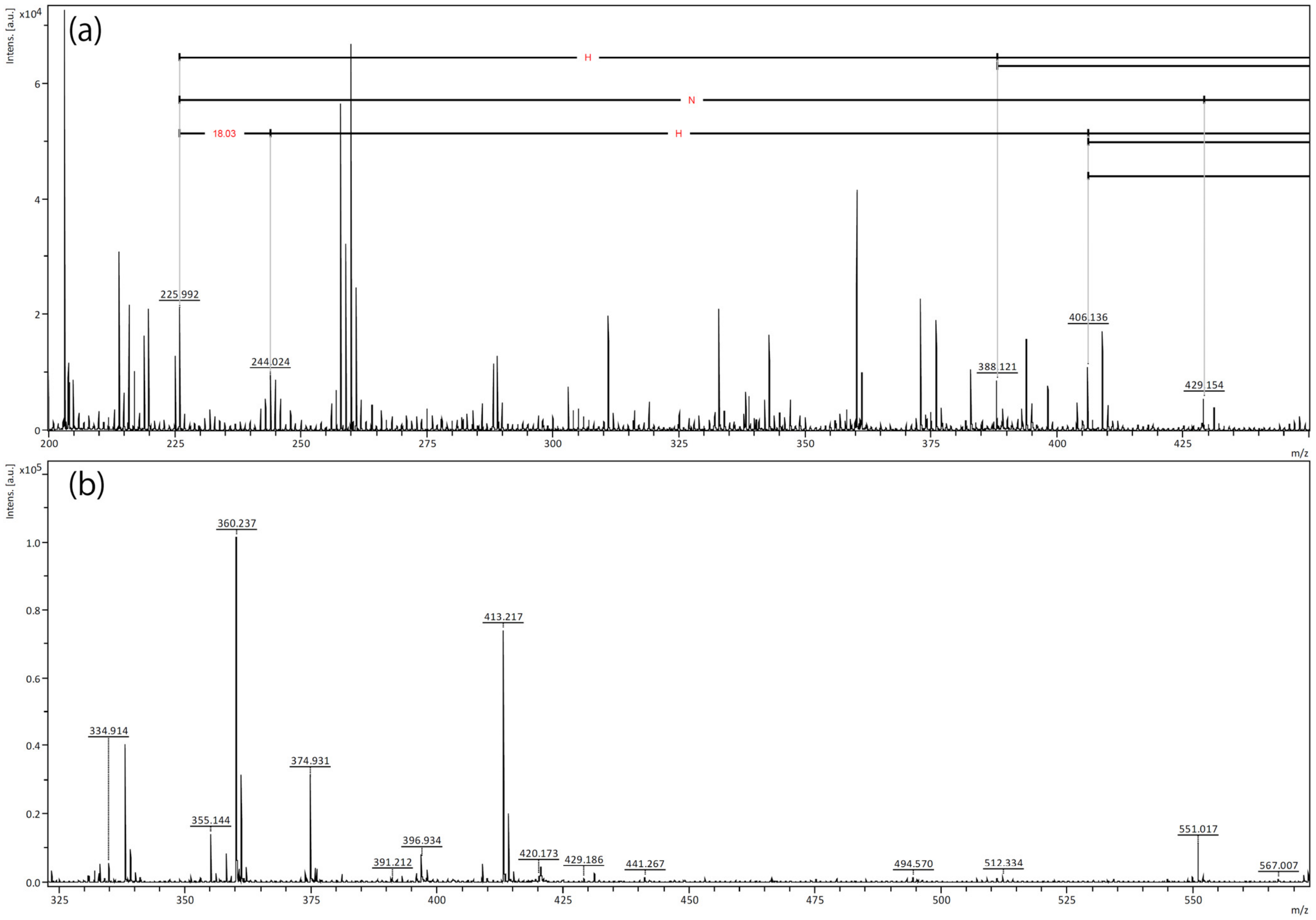

Figure S8. Δ*m/z* 123 comparisons of MALDI mass spectrum of PSM: (a) *m/z* range 200-450 with AHB/Na, (b) *m/z* range 323-473 with BOA/DHB/Na.

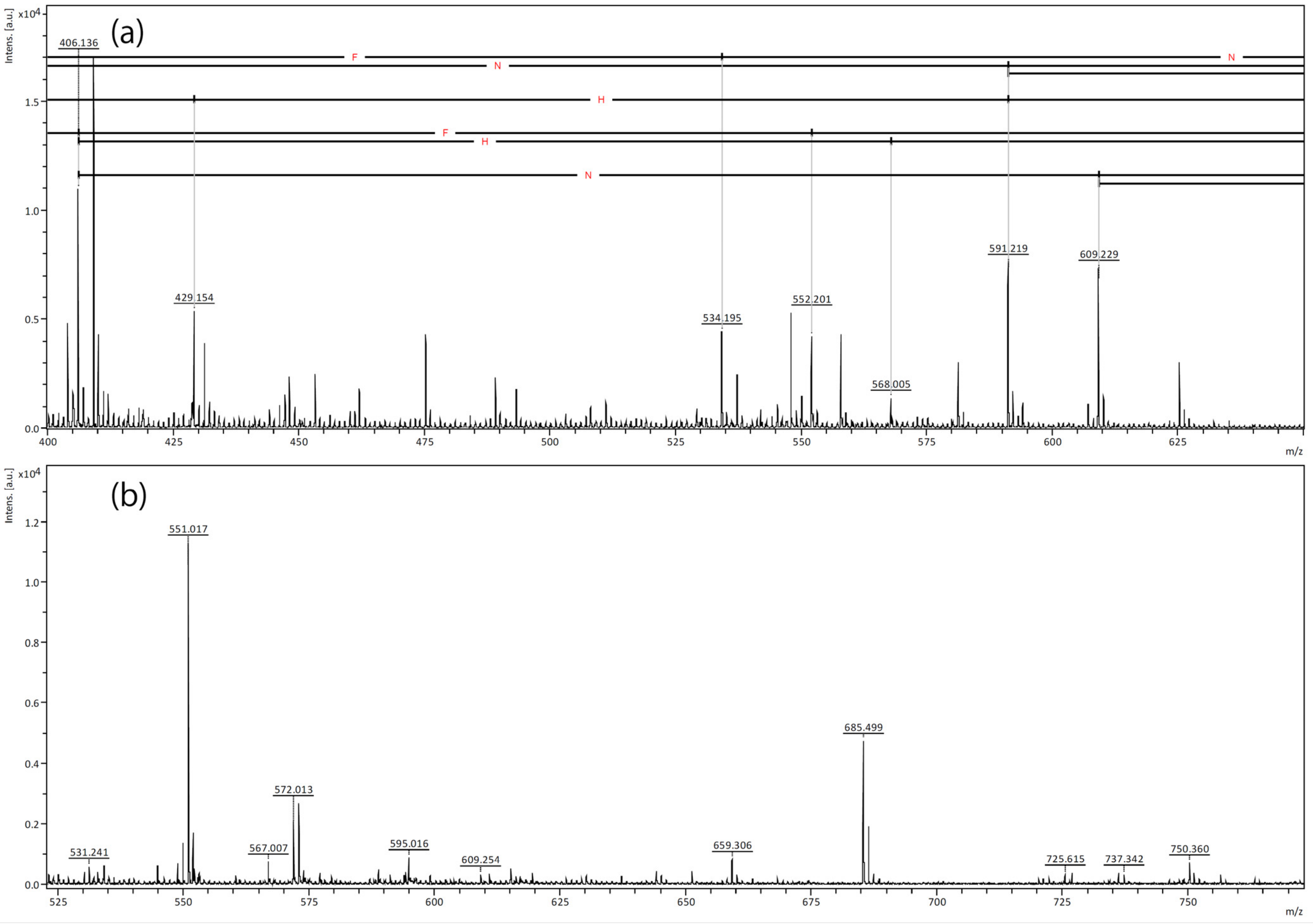

Figure S9. Δ*m/z* 123 comparisons of MALDI mass spectrum of PSM: (a) *m/z* range 400-650 with AHB/Na, (b) *m/z* range 523-773 with BOA/DHB/Na.

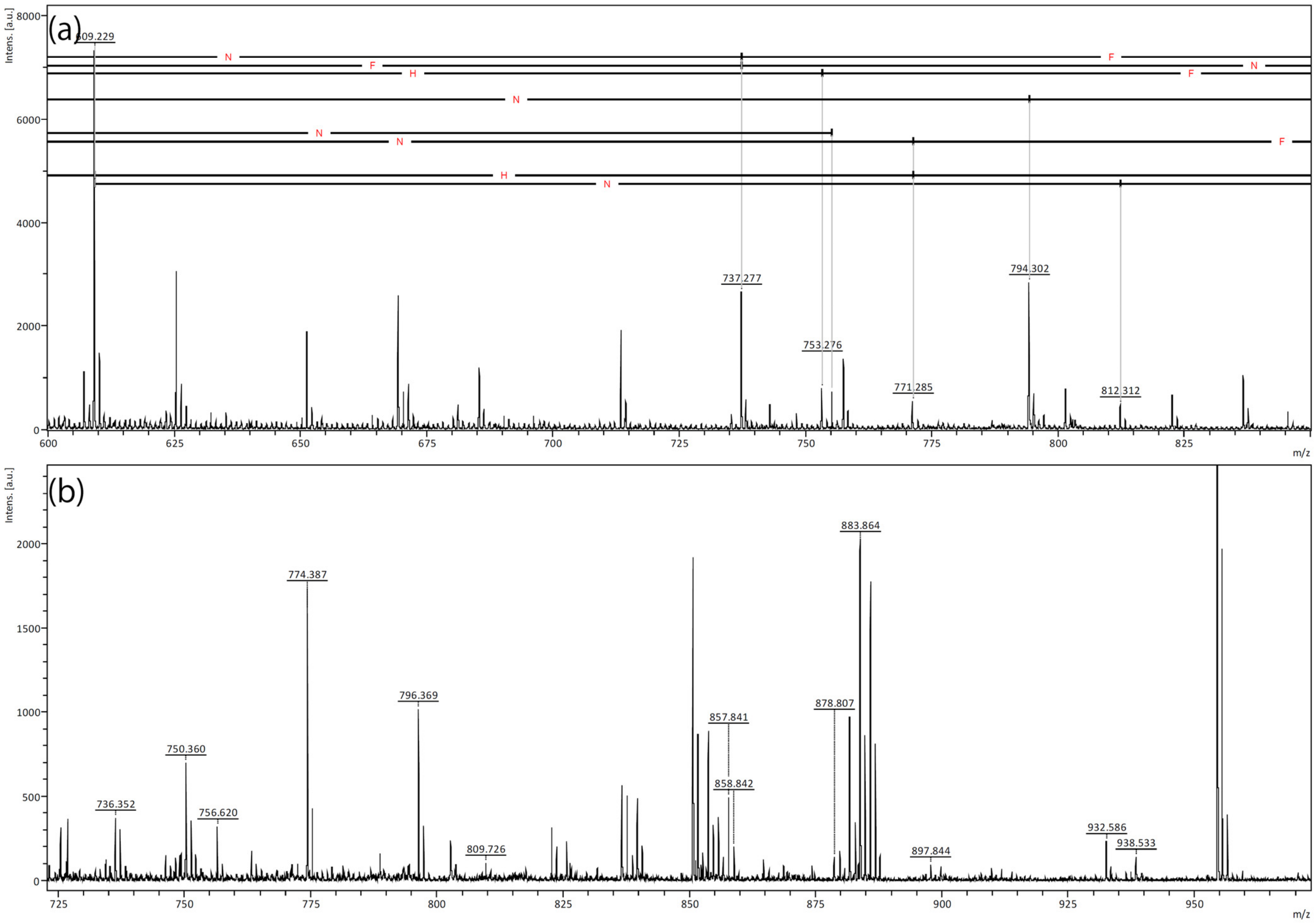

Figure S10. Δ*m/z* 123 comparisons of MALDI mass spectrum of PSM: (a) *m/z* range 600-850 with AHB/Na, (b) *m/z* range 723-973 with BOA/DHB/Na.

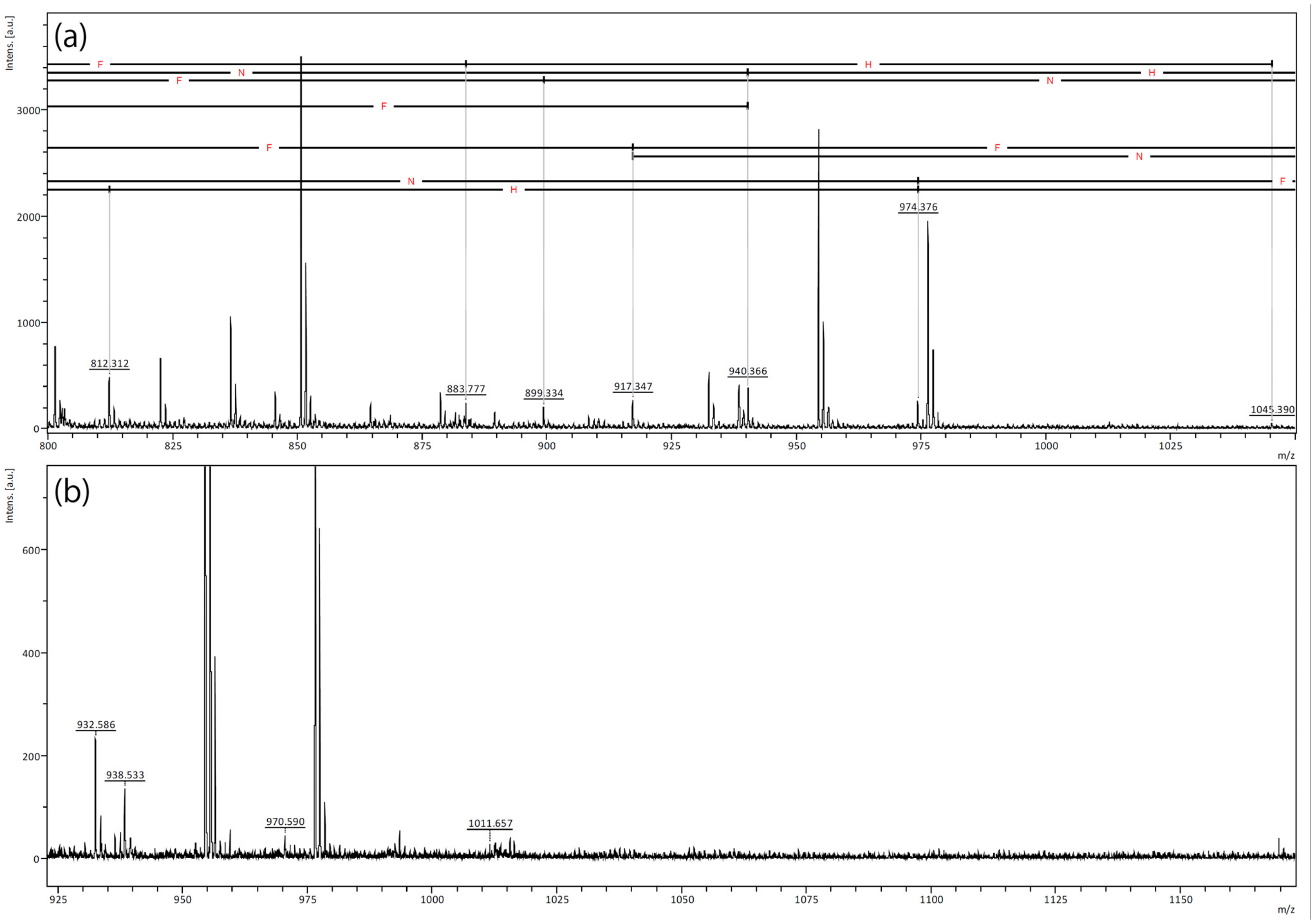

Figure S11. Δ*m/z* 123 comparisons of MALDI mass spectrum of PSM: (a) *m/z* range 800-1050 with AHB/Na, (b) *m/z* range 923-1173 with BOA/DHB/Na.

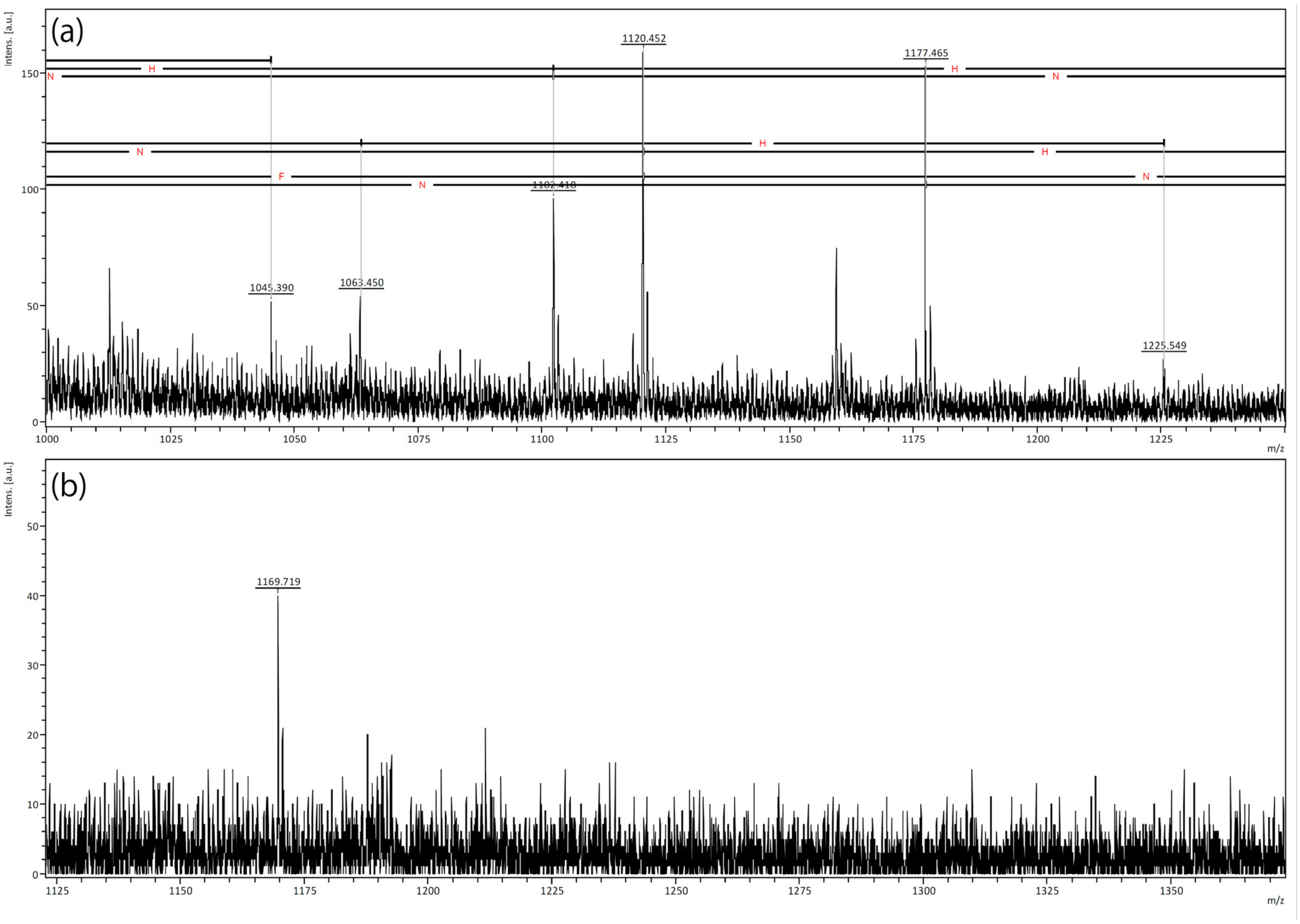

Figure S12. Δ*m/z* 123 comparisons of MALDI mass spectrum of PSM: (a) *m/z* range 1000-1250 with AHB/Na, (b) *m/z* range 1123-1373 with BOA/DHB/Na.

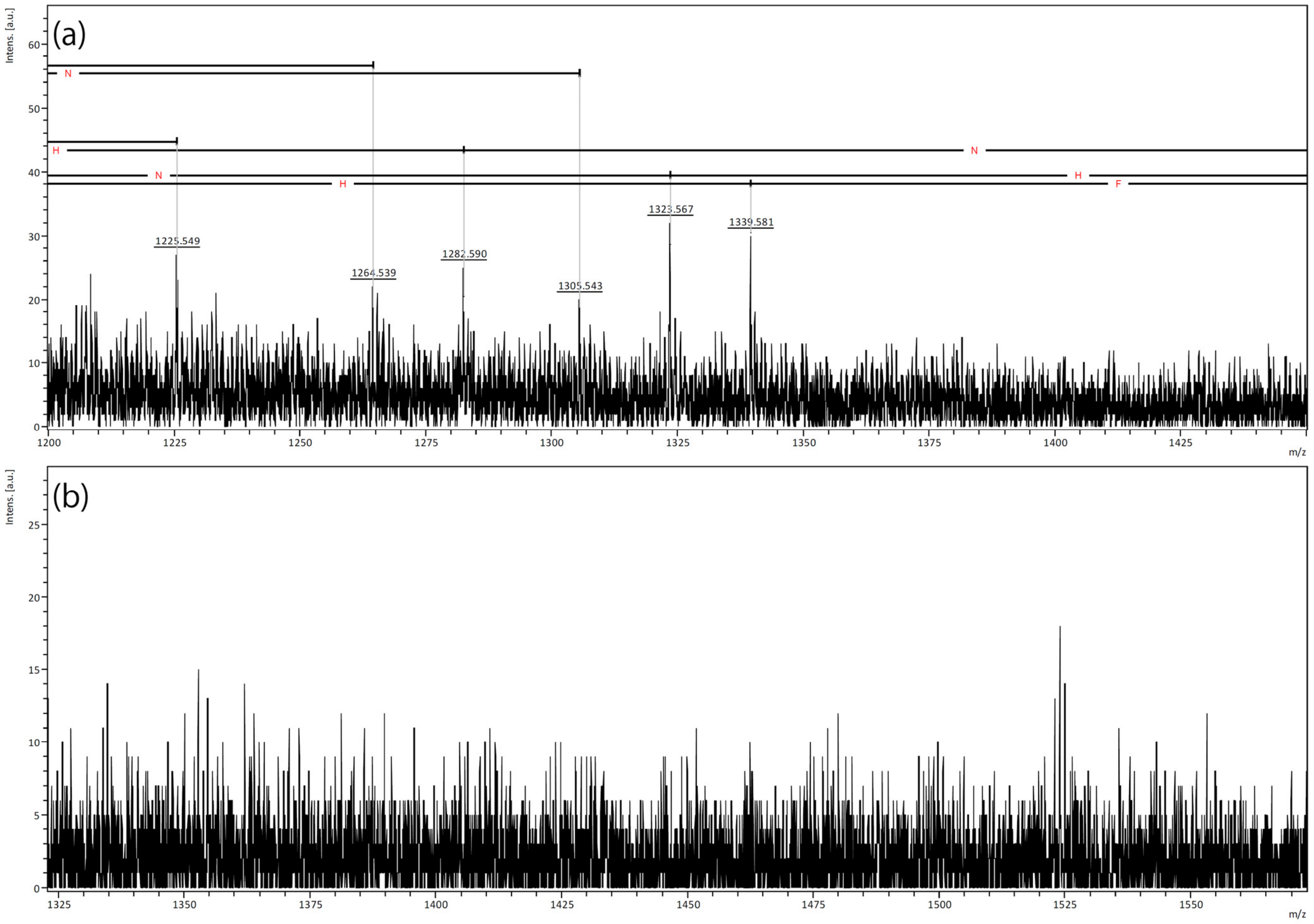

Figure S13. Δ*m/z* 123 comparisons of MALDI mass spectrum of PSM: (a) *m/z* range 1200-1450 with AHB/Na, (b) *m/z* range 1323-1573 with BOA/DHB/Na.

Figure S14~S19. Δ*m/z* 105 comparisons of MALDI mass spectrum of PSM: (a) with AHB/Na, (b) with BOA/DHB/Na. (If the BOA-tagged glycan structure was detected as C ion, the *m/z* difference would be 105. Therefore, the *m/z* range was shifted by 105 for comparison.)

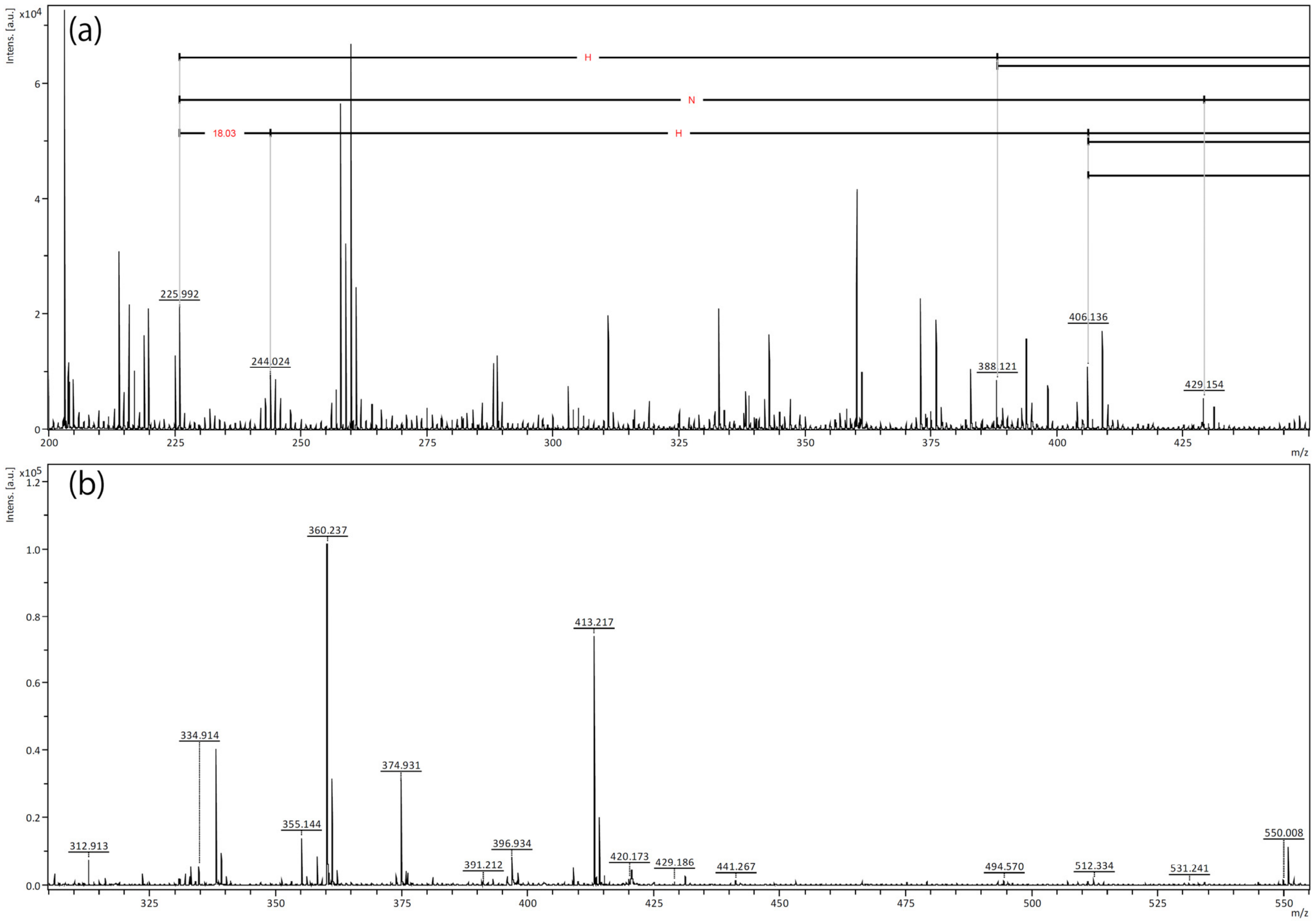

Figure S14. Δ*m/z* 105 comparisons of MALDI mass spectrum of PSM: (a) *m/z* range 200-450 with AHB/Na, (b) *m/z* range 305-555 with BOA/DHB/Na.

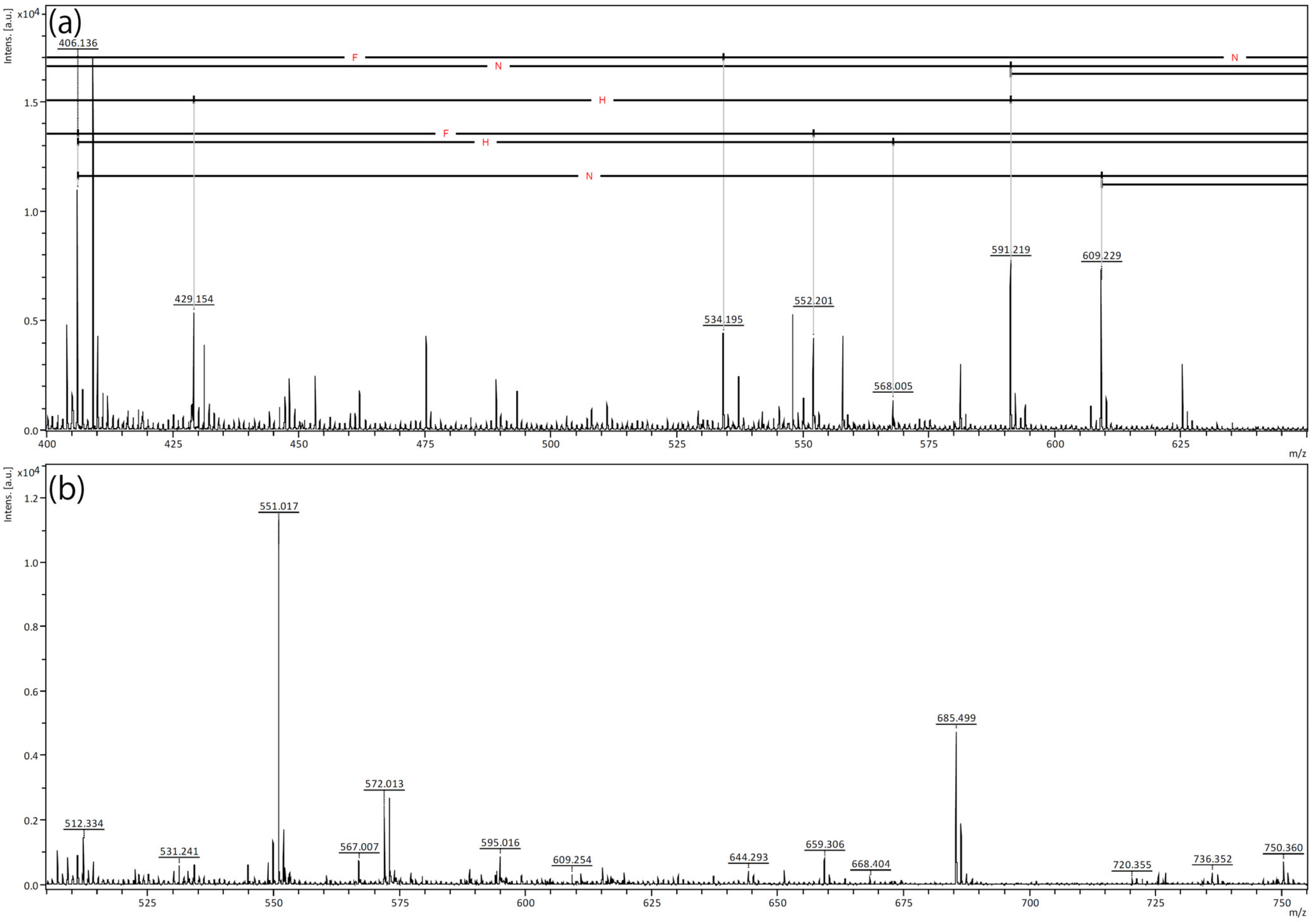

Figure S15. Δ*m/z* 105 comparisons of MALDI mass spectrum of PSM: (a) *m/z* range 400-650 with AHB/Na, (b) *m/z* range 505-755 with BOA/DHB/Na.

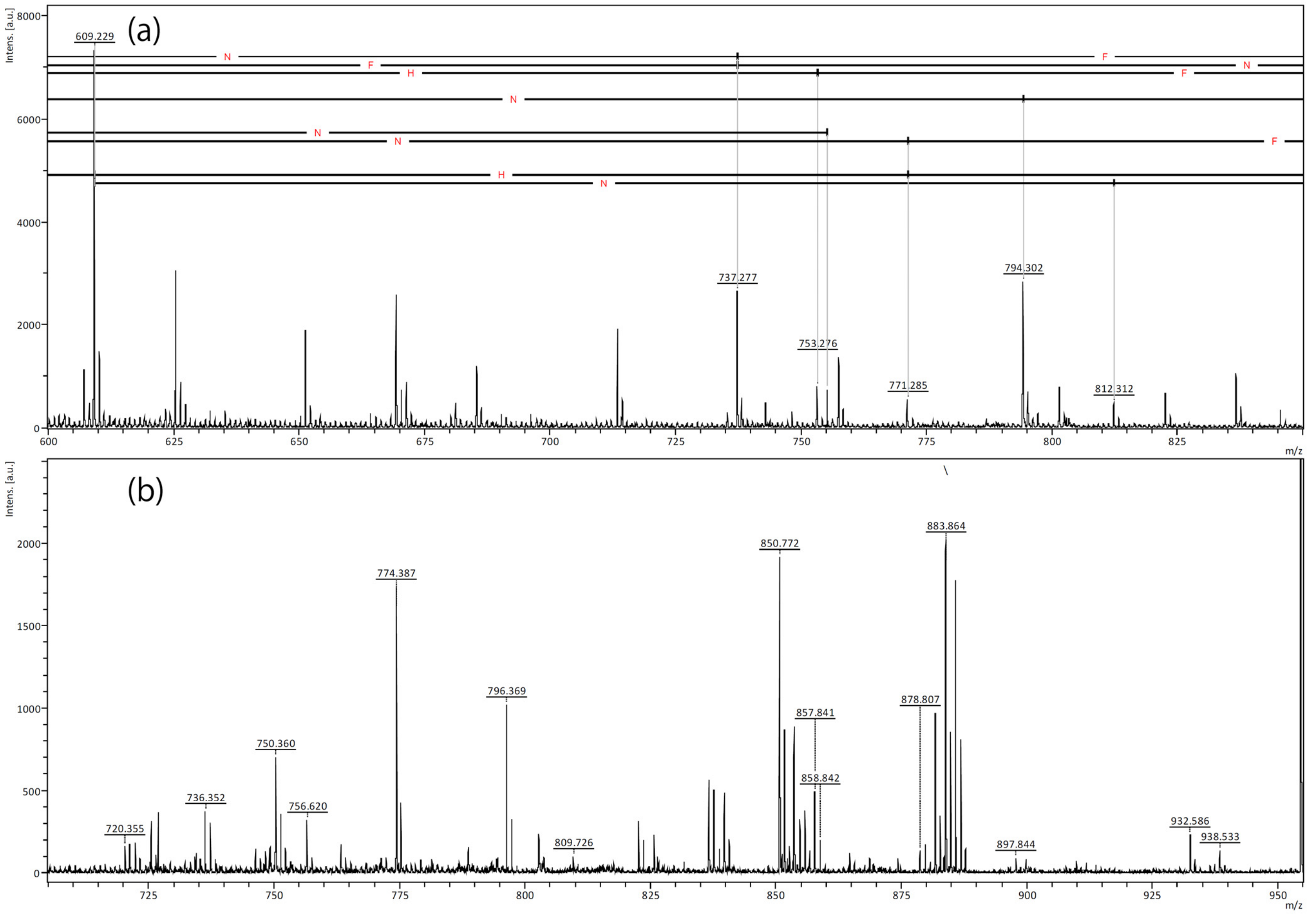

Figure S16. $\Delta m/z$ 105 comparisons of MALDI mass spectrum of PSM: (a) m/z range 600-850 with AHB/Na, (b) m/z range 705-955 with BOA/DHB/Na.

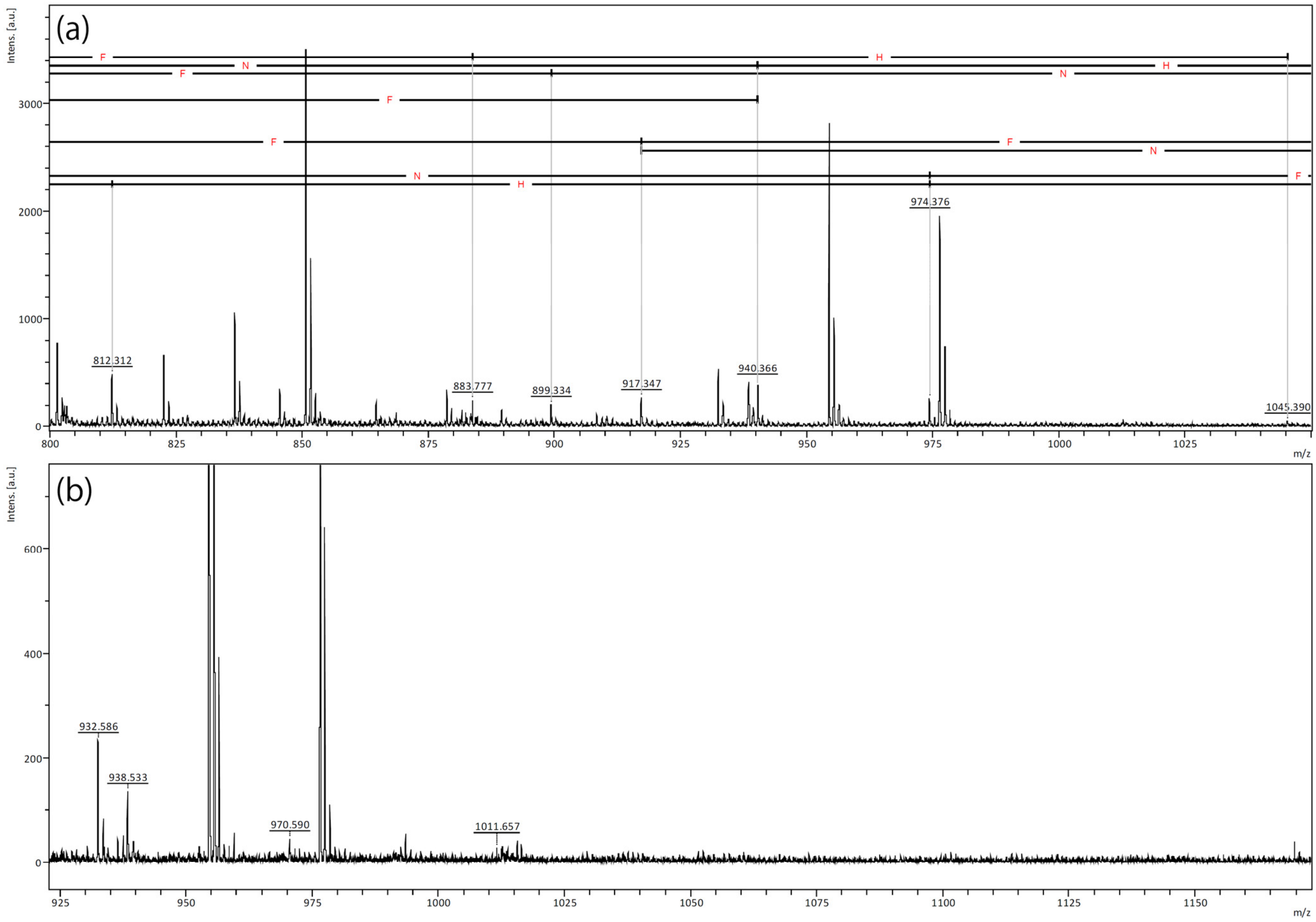

Figure S17. Δm/z 105 comparisons of MALDI mass spectrum of PSM: (a) m/z range 800-1050 with AHB/Na, (b) m/z range 905-1155 with BOA/DHB/Na.

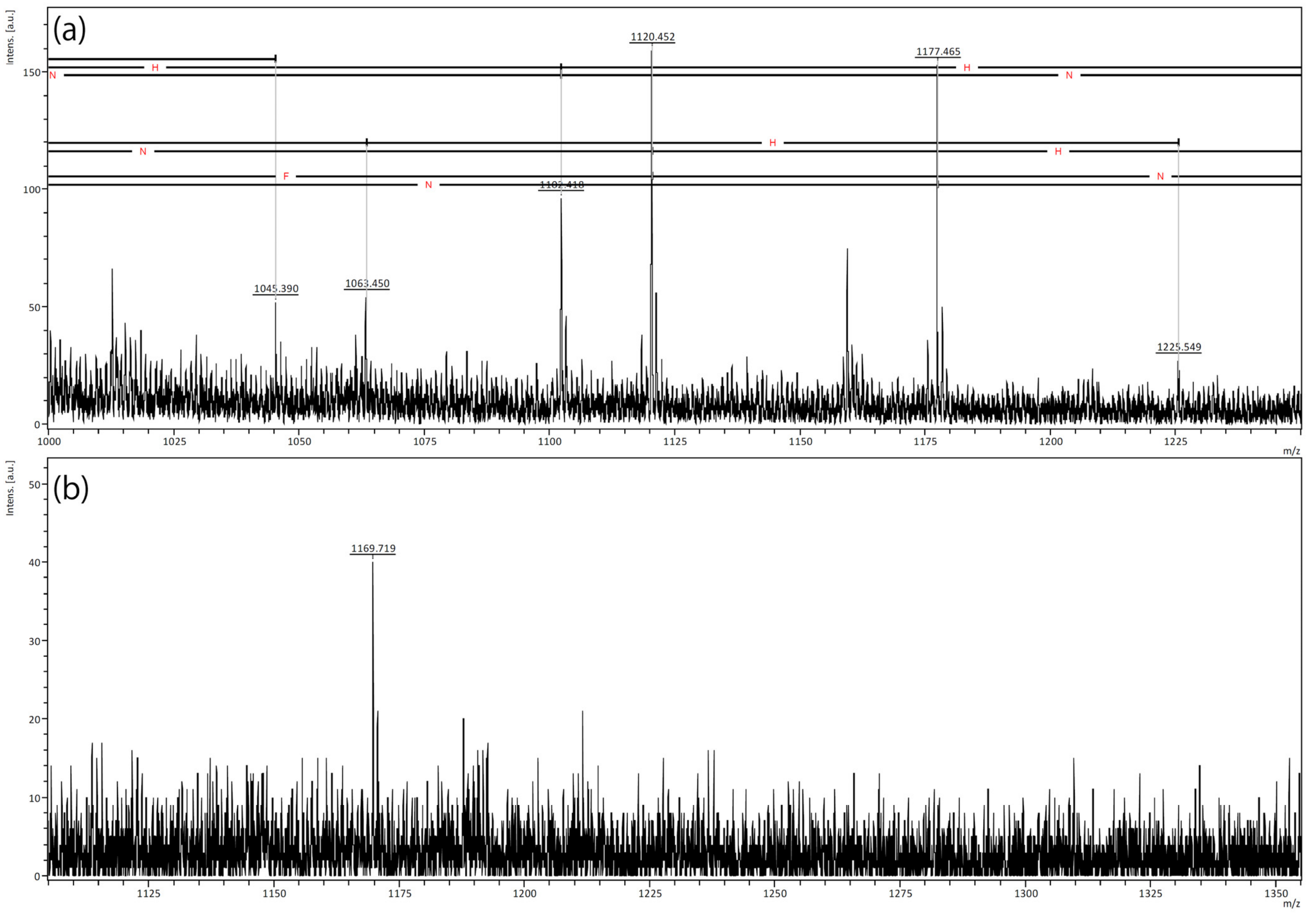

Figure S18. Δ*m/z* 105 comparisons of MALDI mass spectrum of PSM: (a) *m/z* range 1000-1250 with AHB/Na, (b) *m/z* range 1105-1355 with BOA/DHB/Na.

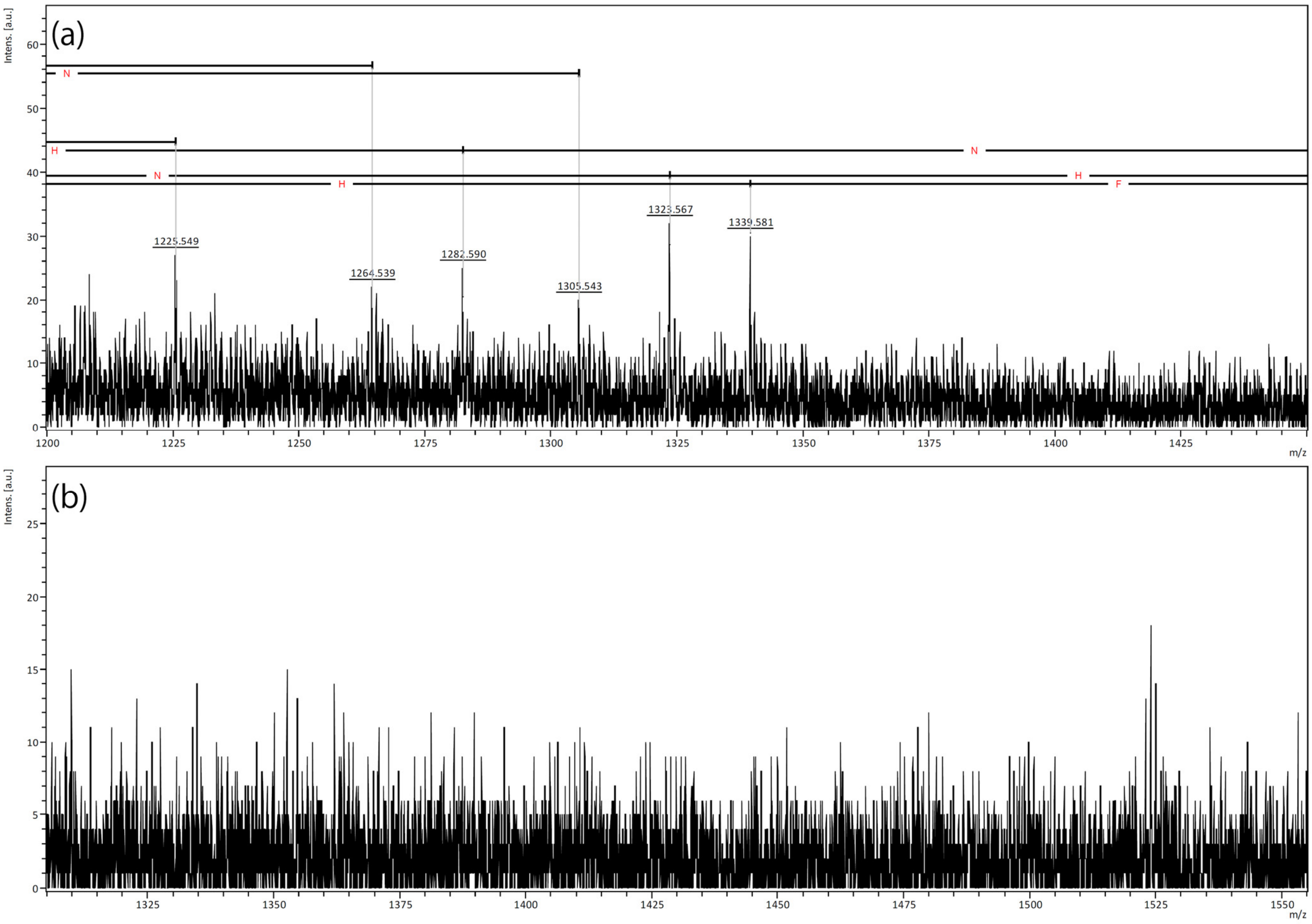

Figure S19. Δ*m*/*z* 105 comparisons of MALDI mass spectrum of PSM: (a) *m*/*z* range 1200-1450 with AHB/Na, (b) *m*/*z* range 1305-1555 with BOA/DHB/Na.

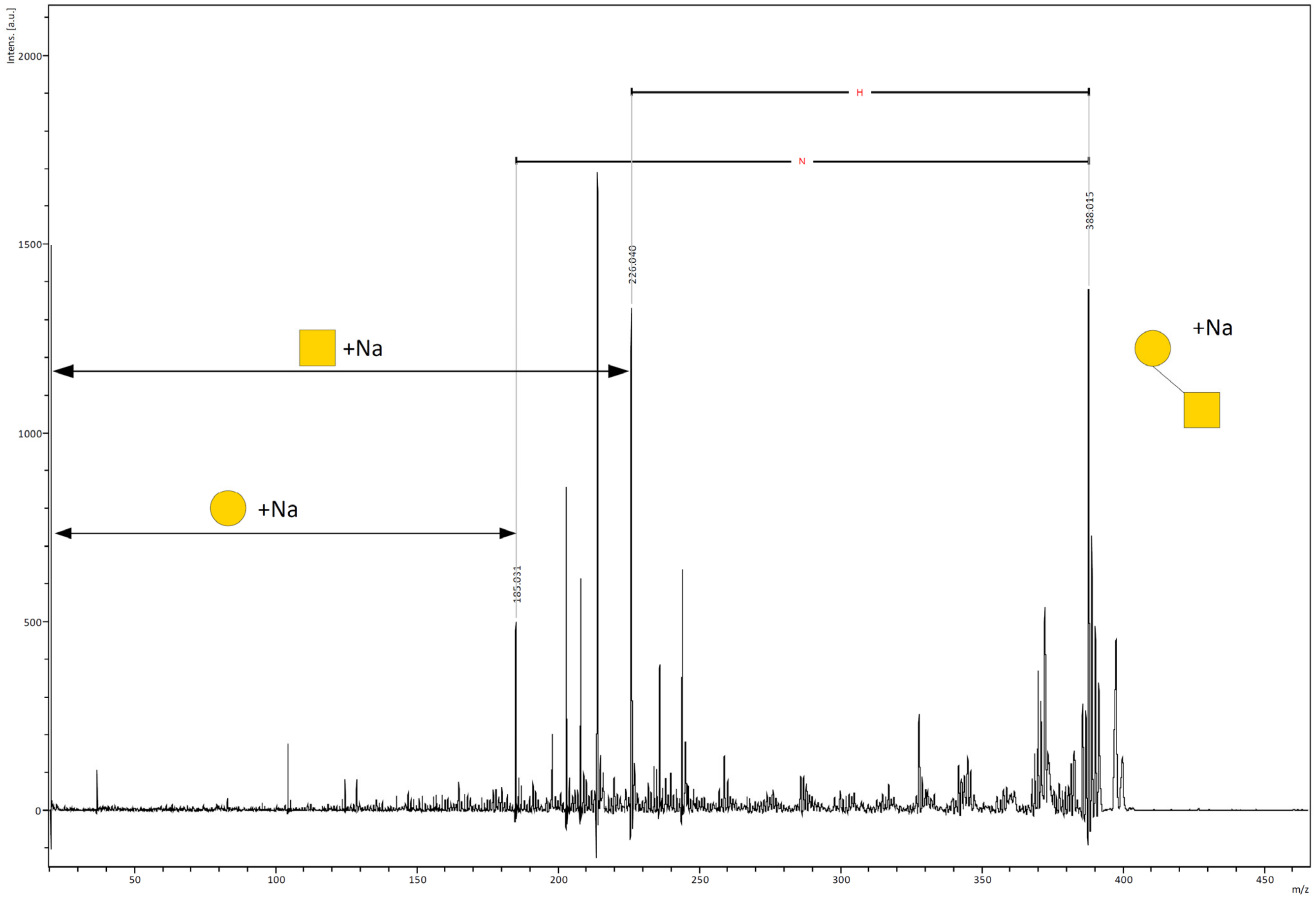

Figure S20. LIFT-TOF/TOF mass spectrum of PSM (1 µgµL^{-1}) with AHB/Na at *m/z* 388 of precursor ion (Table S9)

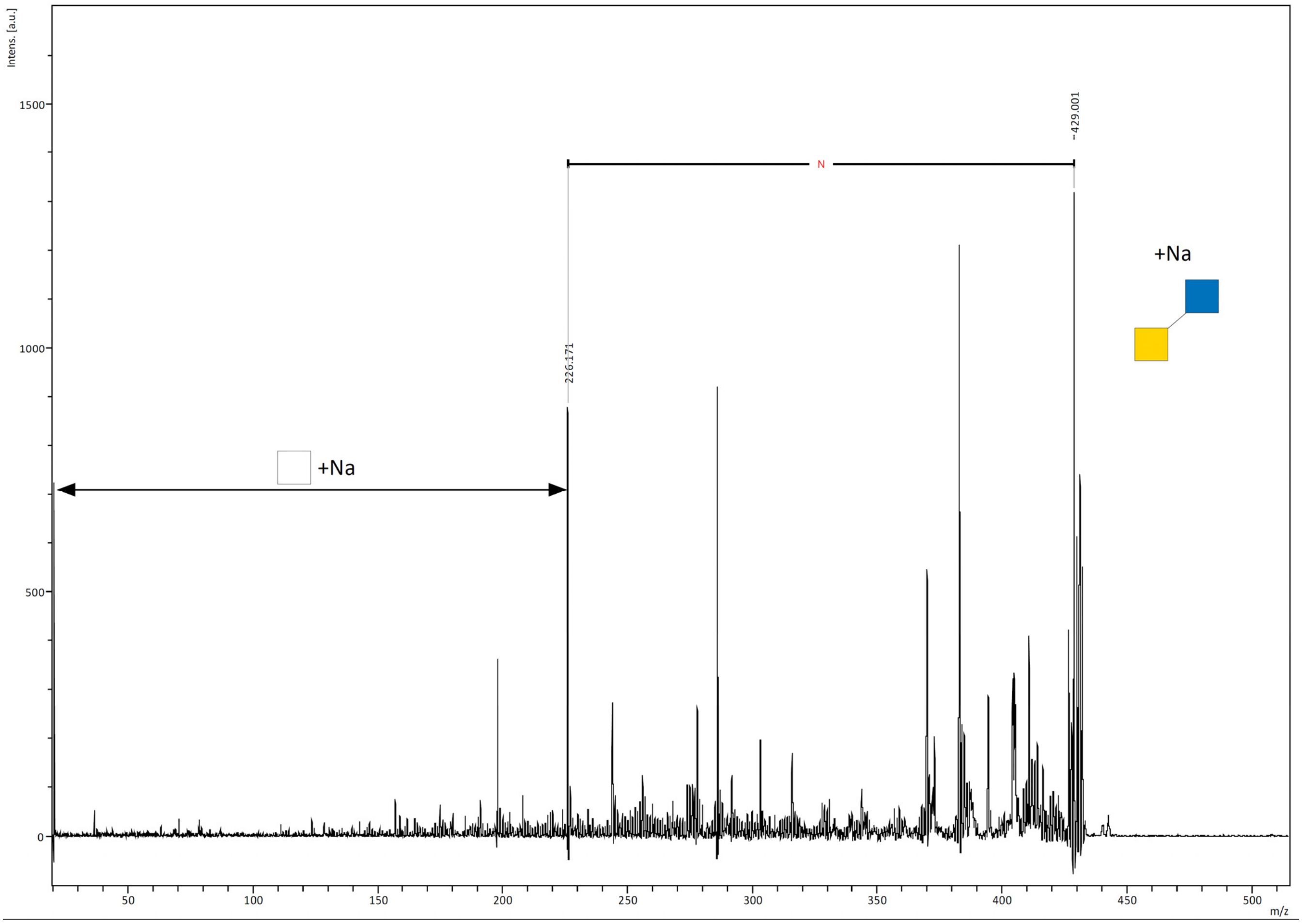

Figure S21. LIFT-TOF/TOF mass spectrum of PSM (1 μgμL^{-1}) with AHB/Na at *m/z* 429 of precursor ion (Table S10)

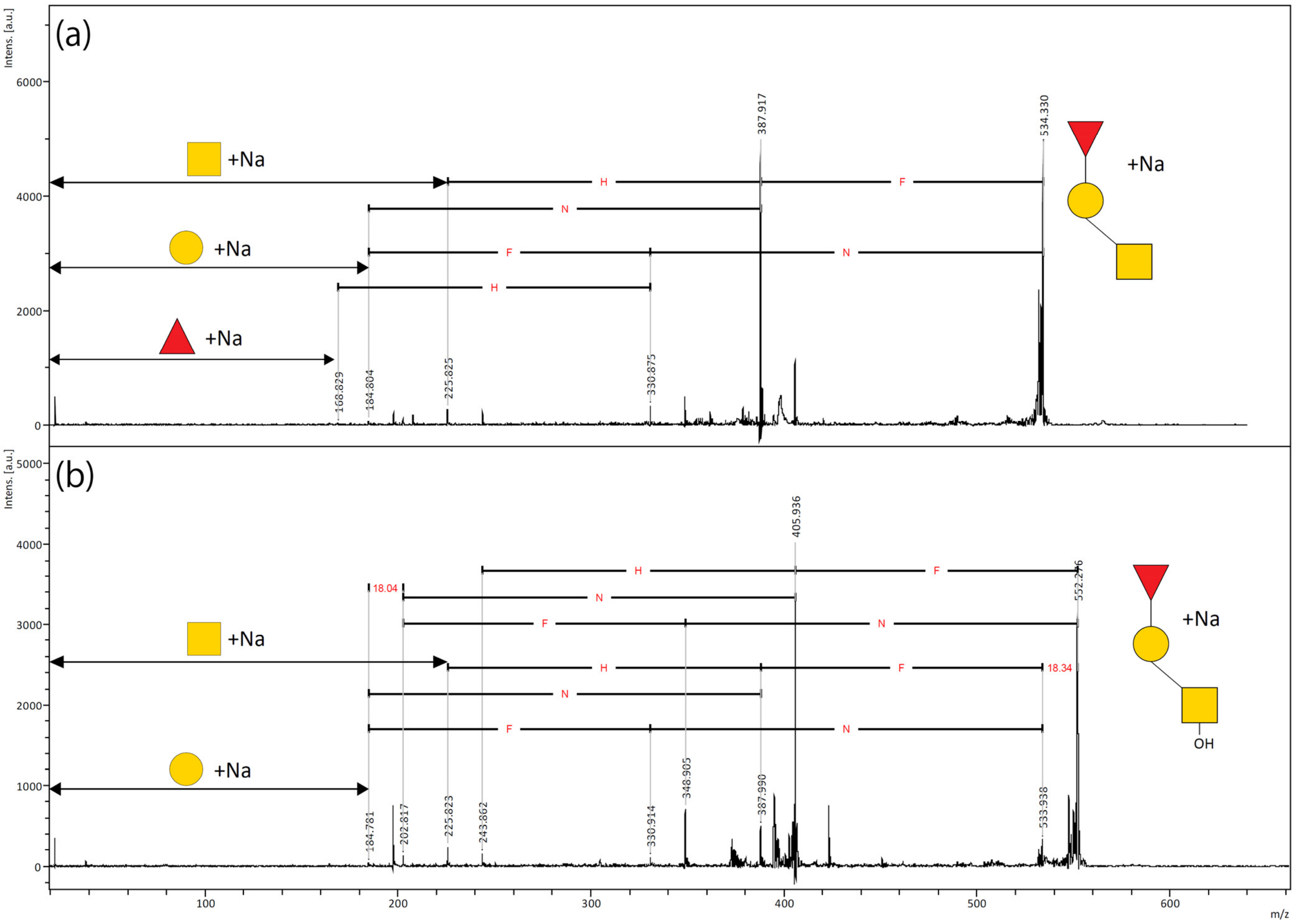

Figure S22. LIFT-TOF/TOF mass spectrum of PSM (1 μgμL^{-1}) with AHB/Na: (a) at *m/z* 534 of precursor ion (Table S11), (b) at *m/z* 552 of precursor ion. (Table S12)

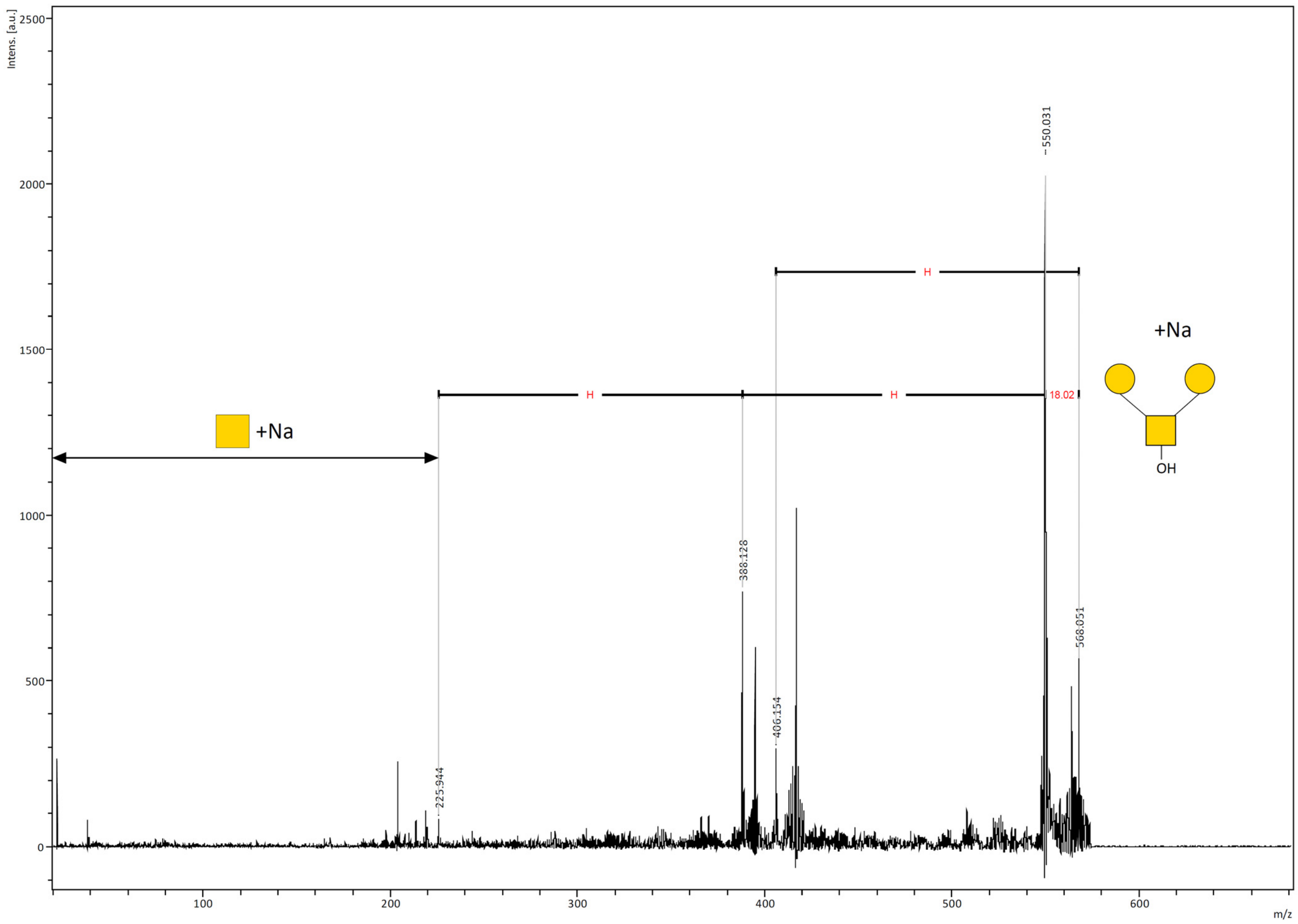

Figure S23. LIFT-TOF/TOF mass spectrum of PSM (1 µgµL^{-1}) with AHB/Na at *m/z* 568 of precursor ion (Table S13),

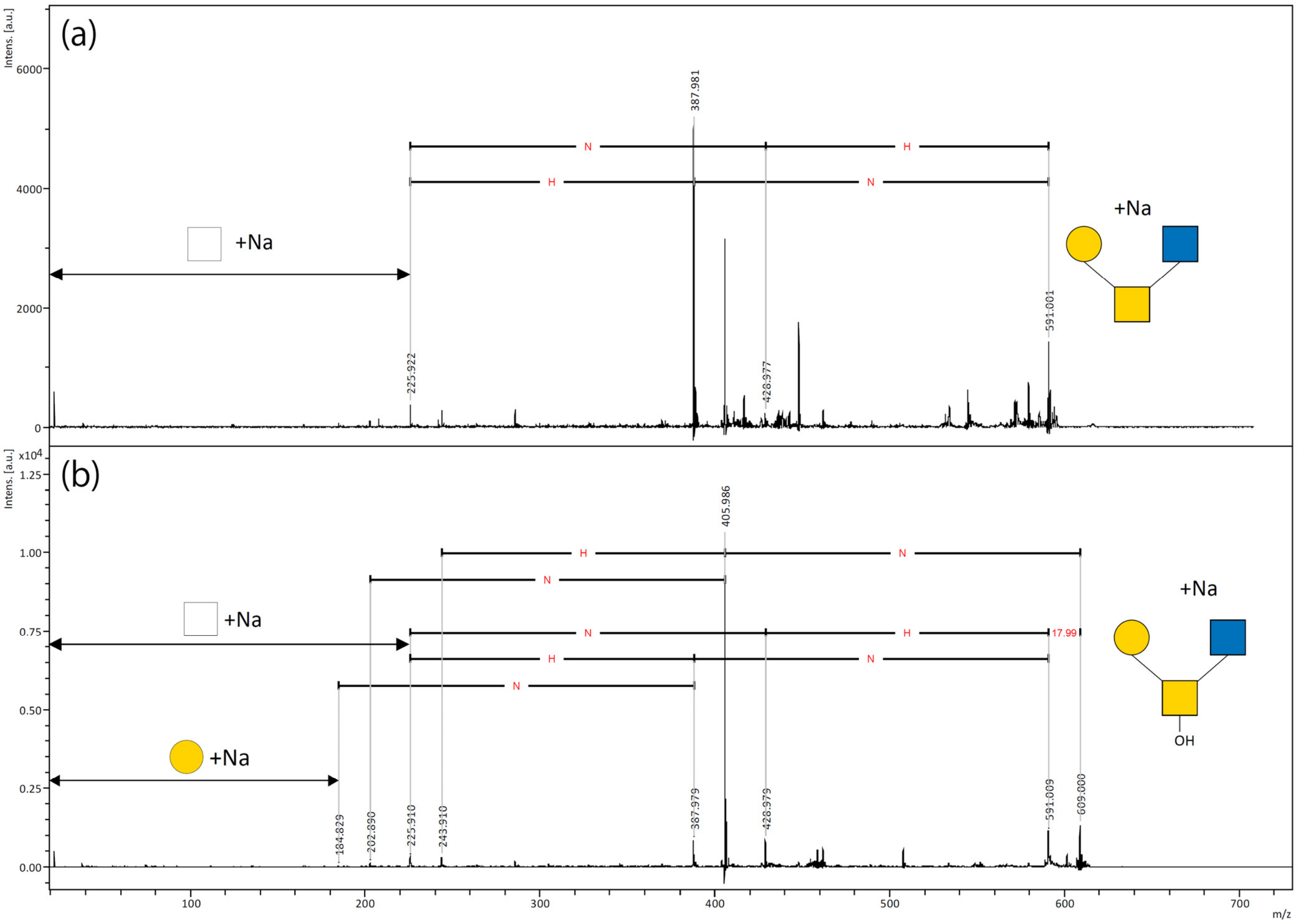

Figure S24. LIFT-TOF/TOF mass spectrum of PSM (1 μgμL^{-1}) with AHB/Na: (a) at *m/z* 591 of precursor ion (Table S14), (b) at *m/z* 609 of precursor ion. (Table S15)

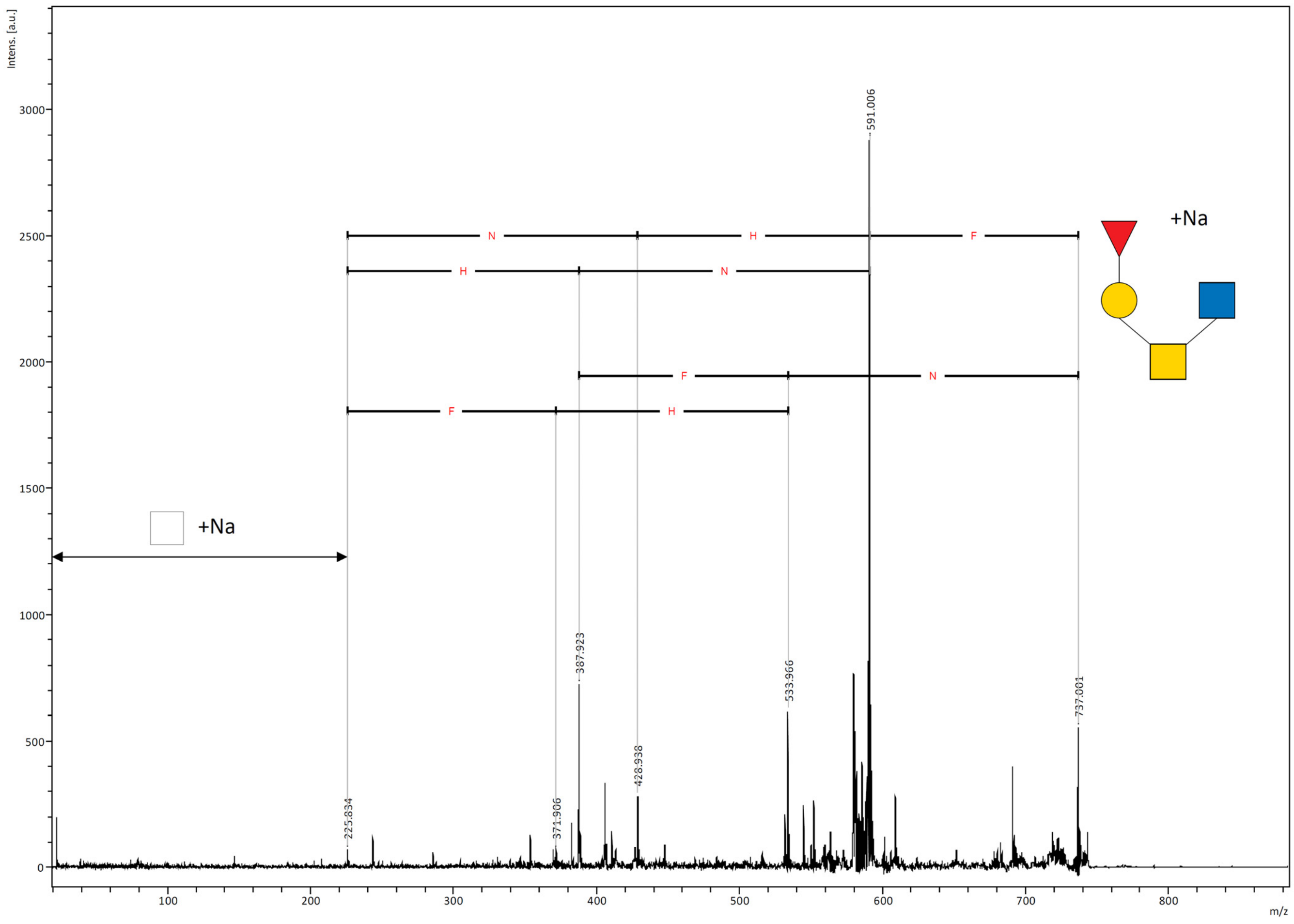

Figure S25. LIFT-TOF/TOF mass spectrum of PSM (1 µgµL^{-1}) with AHB/Na at *m/z* 737 of precursor ion. (Table S16)

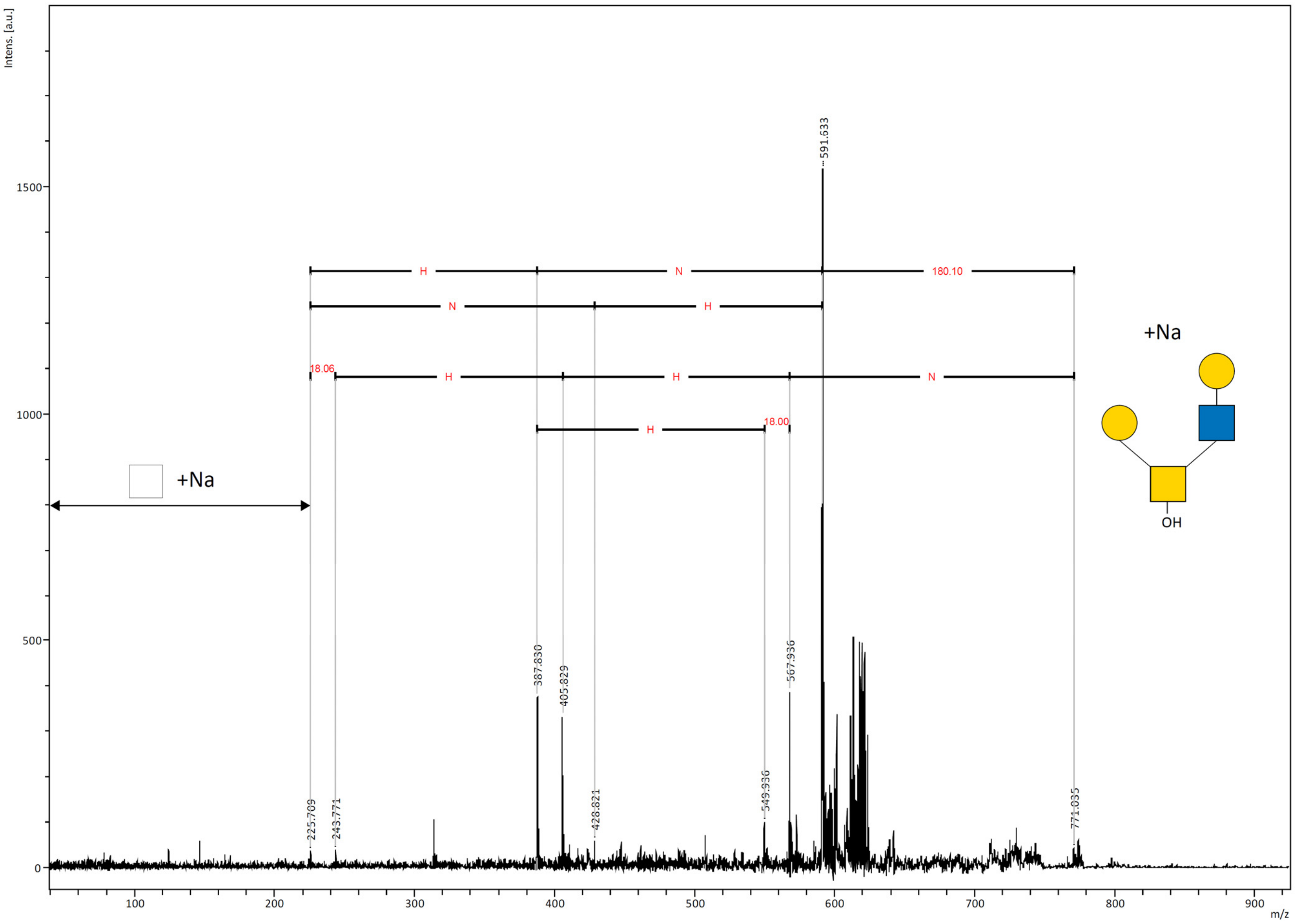

Figure S26. LIFT-TOF/TOF mass spectrum of PSM (1 µgµL^{-1}) with AHB/Na at *m/z* 771 of precursor ion. (Table S17)

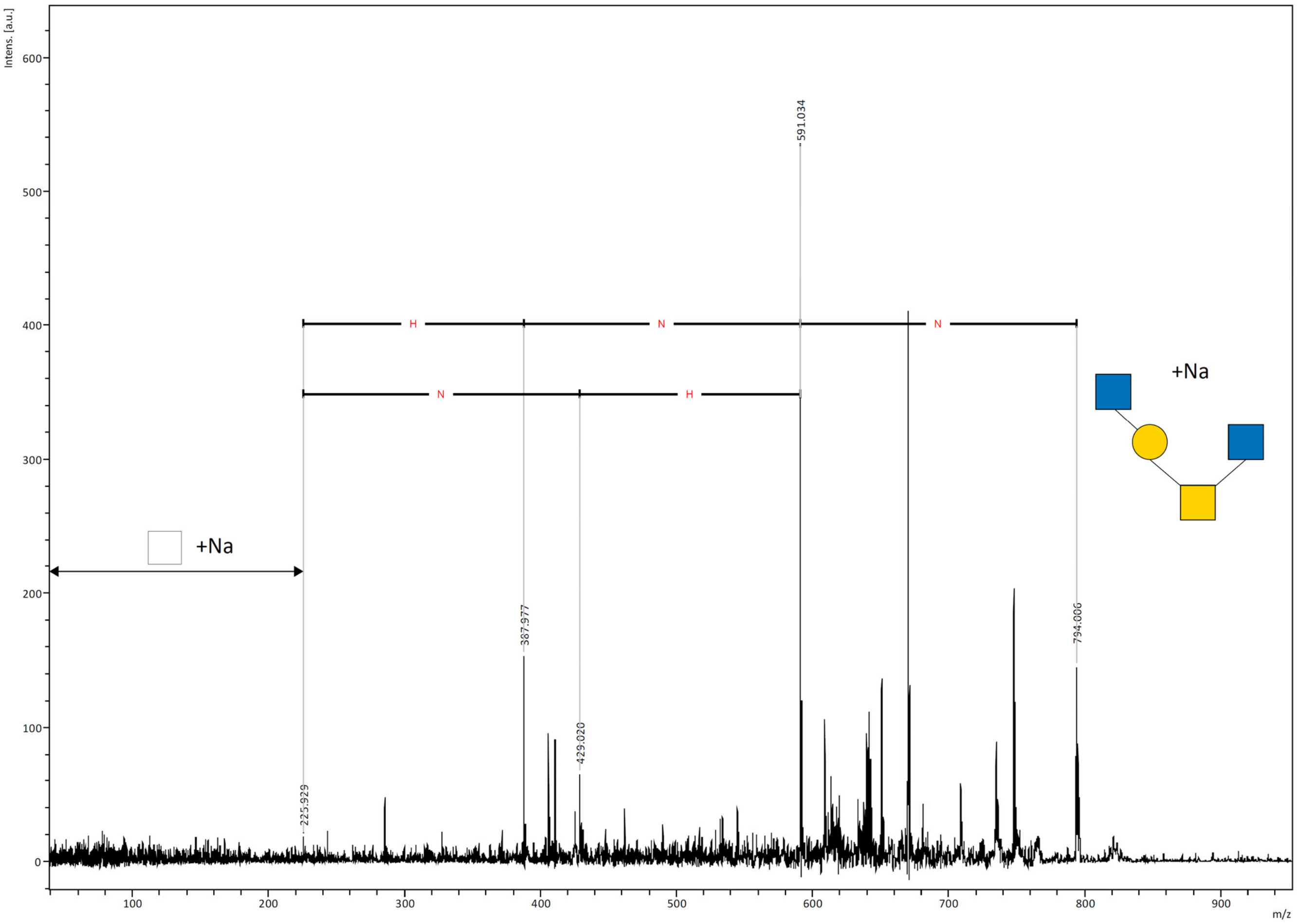

Figure S27. LIFT-TOF/TOF mass spectrum of PSM (1 μgμL^{-1}) with AHB/Na at *m/z* 794 of precursor ion. (Table S18)

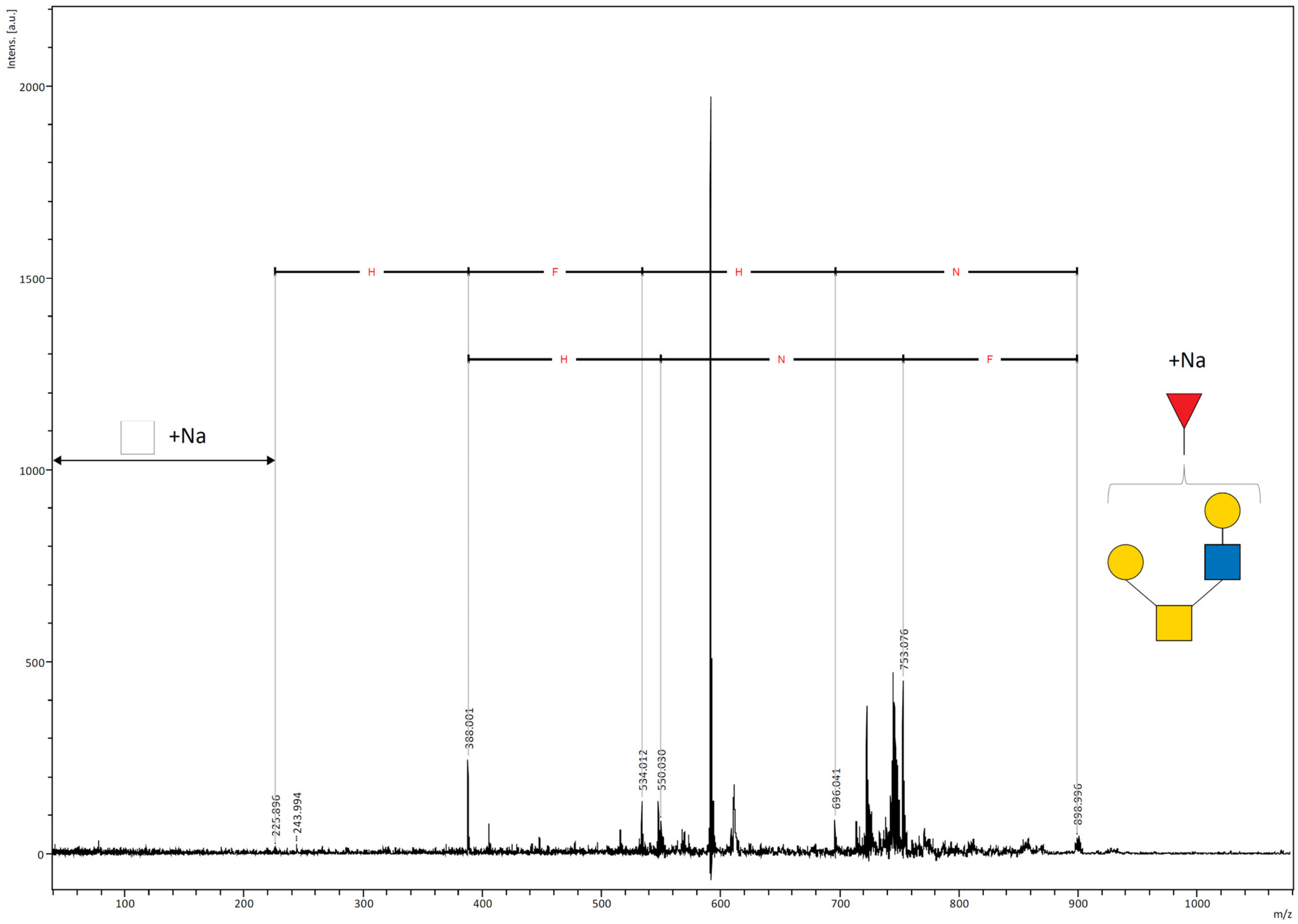

Figure S28. LIFT-TOF/TOF mass spectrum of PSM (1 µgµL^{-1}) with AHB/Na at *m/z* 899 of precursor ion. (Table S19)

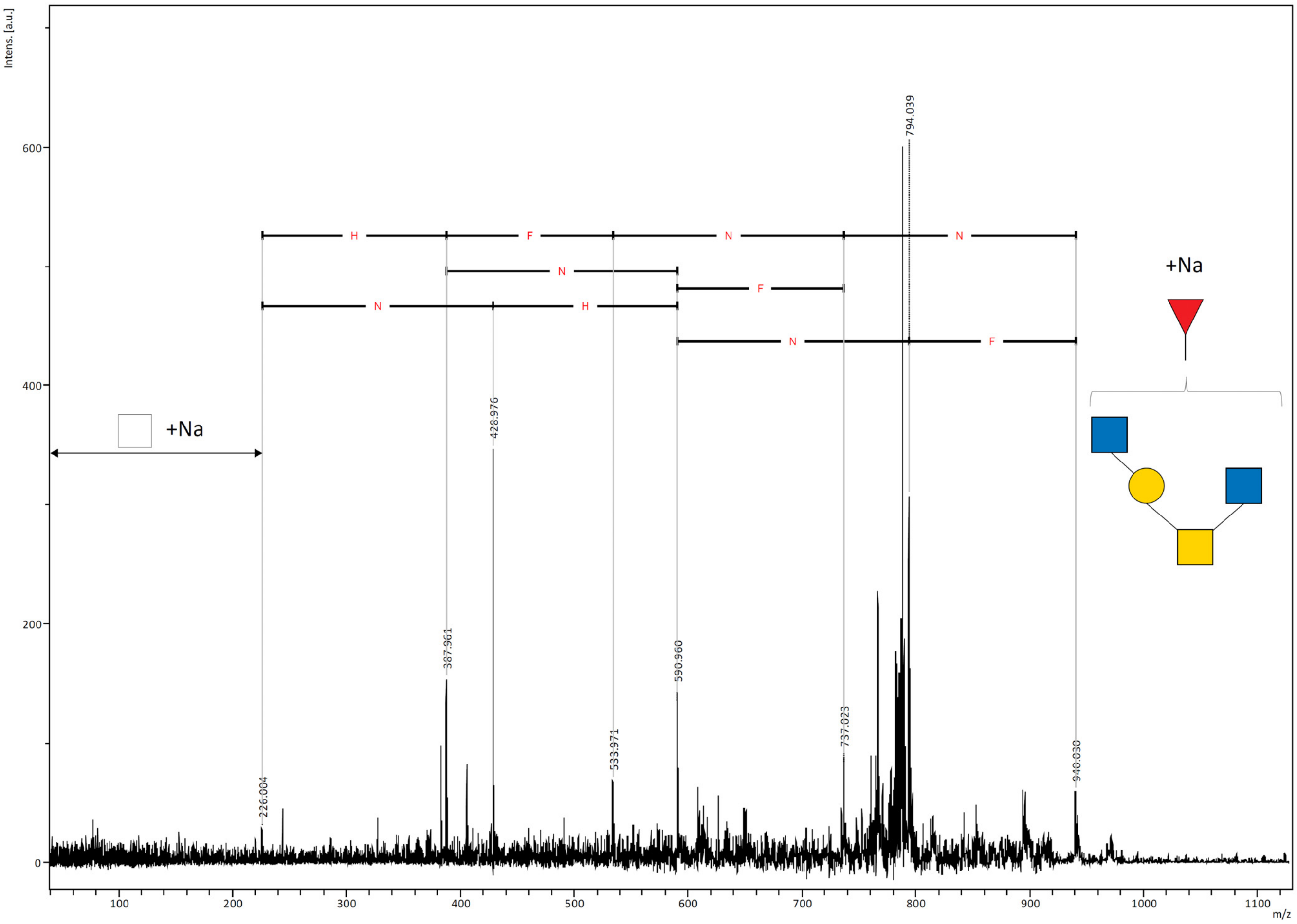

Figure S29. LIFT-TOF/TOF mass spectrum of PSM (1 μgμL^{-1}) with AHB/Na at *m/z* 940 of precursor ion. (Table S20)

SUPPLEMENTARY TABLES

Table S1. List of all ISD products of *O*-glycan from AFGP with DHB in Fig. 3(b).

Structure	Fragment type	Observed *m/z*	Calculated *m/z*	Δ*m/z*	SN ratio
NH	B	388.179	388.12141	0.057	32.910
NH	C	406.206	406.13197	0.074	38.742

Table S2. List of all ISD products of *O*-glycan from AFGP with DAN/DHB/Na in Fig. 3(c).

Structure	Fragment type	Observed *m/z*	Calculated *m/z*	Δ*m/z*	SN ratio
NH	B	388.252	388.12141	0.131	21.352
NH	C	406.281	406.13197	0.149	79.202

Table S3. List of all ISD products of *O*-glycan from AFGP with AHB/Na in Fig. 3(d).

Structure	Fragment type	Observed *m/z*	Calculated *m/z*	Δ*m/z*	SN ratio
NH	B	388.138	388.12141	0.016	151.244
NH	C	406.162	406.13197	0.030	422.628

Table S4. List of *O*-glycan ISD product from PSM with DHB in Fig. S3(a).

Structure	Fragment type	Observed *m/z*	Calculated *m/z*	Δ*m/z*
N	C	244.110	244.07915	0.031
NH	B	388.026	388.12141	0.095
NH	C	406.042	406.13197	0.090
NHF	B	591.114	591.20078	0.087
2NH	C	609.150	609.21134	0.061

Table S5. List of *O*-glycan ISD product from PSM with DAN/DHB/Na in Fig. S3(b).

Structure	Fragment type	Observed *m/z*	Calculated *m/z*	Δ*m/z*
NH	B	388.166	388.12141	0.045
NH	C	406.200	406.13197	0.068
2NH	B	591.296	591.20078	0.095
2NH	C	609.320	609.21134	0.109
2NHF	B	737.366	737.25869	0.107
3NH	B	794.412	794.28015	0.132
3N2H	C	974.537	974.34353	0.193
3N2HF	B	1102.596	1102.39088	0.205
4N2H	C	1177.553	1177.42290	0.130

Table S6. List of *O*-glycan ISD product from PSM with AHB/Na in Fig. 4.

Structure	Fragment type	Observed *m/z*	Calculated *m/z*	Δ*m/z*
N	B	225.992	226.06859	0.077
N	C	244.024	244.07915	0.056
NH	B	388.121	388.12141	0.000
NH	C	406.136	406.13197	0.004
2N	B	429.154	429.14796	0.006
NHF	B	534.195	534.17932	0.016
NHF	C	552.201	552.18988	0.012
2NH	B	591.219	591.20078	0.019
2NH	C	609.229	609.21134	0.017
2NHF	B	737.277	737.25869	0.019
2N2H	B	753.276	753.25360	0.022
2NHF	C	755.280	755.26925	0.011
2N2H	C	771.285	771.26416	0.021
3NH	B	794.302	794.28015	0.022
3NH	C	812.312	812.29071	0.021
2N2HF	B	899.334	899.31151	0.023
2N2HF	C	917.347	917.32207	0.025
3NHF	B	940.366	940.33806	0.028
3N2H	C	974.376	974.34353	0.032
2N2H2F	B	1045.390	1045.36942	0.021
2N2H2F	C	1063.450	1063.37998	0.070
3N2HF	B	1102.418	1102.39088	0.027
3N2HF	C	1120.452	1120.40144	0.051
4N2H	B	1159.460	1159.41234	0.048
4N2H	C	1177.465	1177.42290	0.042
2N3H2F	C	1225.549	1225.43280	0.116
3N3HF	B	1264.539	1264.44370	0.095
3N3HF	C	1282.590	1282.45426	0.136
4N2HF	B	1305.543	1305.47025	0.072
4N2HF	C	1323.567	1323.48081	0.086
4N3H	C	1339.581	1339.47572	0.105
4N3HF	C	1485.731	1485.53363	0.197

Table S7. List of product ion from *O*-glycan ISD product (*m/z* 406) from cAFGP with AHB/Na in Fig. 5(a).

**Structure**	**Fragment type**	**Observed *m/z***	**Calculated *m/z***	**Δ*m/z***
H	B	185.118	185.04204	0.076
H	C	203.139	203.0526	0.086
N	B	226.146	226.06859	0.078
N	C	244.148	244.07915	0.069
NH	B	387.989	388.12141	0.132
NH	C	406.000	406.13197	0.132

Table S8. List of product ion from *O*-glycan ISD product (*m/z* 406) from PSM with AHB/Na in Fig. 5(b).

**Structure**	**Fragment type**	**Observed *m/z***	**Calculated *m/z***	**Δ*m/z***
H	B	185.090	185.04204	0.048
H	C	203.109	203.05260	0.056
N	B	226.114	226.06859	0.046
N	C	244.102	244.07915	0.023
NH	B	387.962	388.12141	0.159
NH	C	405.998	406.13197	0.134

Table S9. List of product ion from *O*-glycan ISD product (*m/z* 388) from PSM with AHB/Na in Fig. S20.

Structure	Fragment type	Observed *m/z*	Calculated *m/z*	Δ*m/z*
H	B	185.031	185.04204	0.011
N	B	226.040	226.06859	0.028
NH	B	388.015	388.12141	0.107

Table S10. List of product ion from *O*-glycan ISD product (*m/z* 429) from PSM with AHB/Na in Fig. S21.

Structure	Fragment type	Observed *m/z*	Calculated *m/z*	Δ*m/z*
N	B	226.171	226.06859	0.102
2N	B	429.001	429.14796	0.147

Table S11. List of product ion from *O*-glycan ISD product (*m/z* 534) from PSM with AHB/Na in Fig. S22(a).

Structure	Fragment type	Observed *m/z*	Calculated *m/z*	Δ*m/z*
F	B	168.829	169.04713	0.218
H	B	184.804	185.04204	0.238
N	B	225.825	226.06859	0.243
HF	B	330.875	331.0.9995	0.225
NH	B	387.917	388.12141	0.205
NHF	B	534.330	534.17932	0.151

Table S12. List of product ion from *O*-glycan ISD product (*m/z* 552) from PSM with AHB/Na in Fig. S22(b).

Structure	Fragment type	Observed *m/z*	Calculated *m/z*	Δ*m/z*
H	B	184.781	185.04204	0.261
H	C	202.817	203.05260	0.235
N	B	225.823	226.06859	0.245
N	C	243.862	244.07915	0.217
HF	B	330.914	331.09995	0.186
HF	C	348.905	349.11051	0.206
NH	B	387.990	388.12141	0.132
NH	C	405.936	406.13197	0.196
NHF	B	533.938	534.17932	0.242
NHF	C	552.276	552.18988	0.086

Table S13. List of product ion from *O*-glycan ISD product (*m/z* 568) from PSM with AHB/Na in Fig. S23.

Structure	Fragment type	Observed *m/z*	Calculated *m/z*	Δ*m/z*
N	B	225.944	226.06859	0.125
NH	B	388.128	388.12141	0.007
NH	C	406.154	406.13197	0.022
N2H	B	550.031	550.17423	0.143
N2H	C	568.051	568.18479	0.134

Table S14. List of product ion from *O*-glycan ISD product (*m/z* 591) from PSM with AHB/Na in Fig. S24(a).

Structure	Fragment type	Observed *m/z*	Calculated *m/z*	Δ*m/z*
N	B	225.922	226.06859	0.146
NH	B	387.981	388.12141	0.140
2N	B	428.977	429.14796	0.171
2NH	B	591.001	591.20078	0.200

Table S15. List of product ion from *O*-glycan ISD product (*m/z* 609) from PSM with AHB/Na in Fig. S24(b).

Structure	Fragment type	Observed *m/z*	Calculated *m/z*	Δ*m/z*
H	B	184.829	185.04204	0.213
H	C	202.890	203.05260	0.162
N	B	225.910	226.06859	0.158
N	C	243.910	244.07915	0.169
NH	B	387.979	388.12141	0.143
NH	C	405.986	406.13197	0.146
2N	B	428.979	429.14796	0.169
2N	C	446.957	447.15852	0.202
2NH	B	591.009	591.20078	0.192
2NH	C	609.000	609.21134	0.212

Table S16. List of product ion from *O*-glycan ISD product (*m/z* 737) from PSM with AHB/Na in Fig. S25.

Structure	Fragment type	Observed *m/z*	Calculated *m/z*	Δ*m/z*
N	B	225.834	226.06859	0.235
NH	B	387.923	388.12141	0.198
2N	B	428.938	429.14796	0.210
NHF	B	533.966	534.17932	0.214
2NH	B	591.006	591.20078	0.195
2NHF	B	737.001	737.25869	0.257

Table S17. List of product ion from *O*-glycan ISD product (*m/z* 771) from PSM with AHB/Na in Fig. S26.

Structure	Fragment type	Observed *m/z*	Calculated *m/z*	Δ*m/z*
N	B	225.709	226.06859	0.359
N	C	243.771	244.07915	0.308
NH	B	387.830	388.12141	0.291
NH	C	405.829	406.13197	0.303
2N	B	428.821	429.14796	0.327
N2H	B	549.936	550.17423	0.238
N2H	C	567.936	568.18479	0.249
2NH	B	590.938	591.20078	0.263
2NH	C	609.002	609.21134	0.209
2N2H	C	771.035	771.26416	0.229

Table S18. List of product ion from *O*-glycan ISD product (*m/z* 794) from PSM with AHB/Na in Fig. S27.

Structure	Fragment type	Observed *m/z*	Calculated *m/z*	Δ*m/z*
N	B	225.929	226.06859	0.139
NH	B	387.977	388.12141	0.145
2N	B	429.020	429.14796	0.128
2NH	B	591.034	591.20078	0.167
3NH	B	794.006	794.28015	0.274

Table S19. List of product ion from *O*-glycan ISD product (*m/z* 899) from PSM with AHB/Na in Fig. S28.

Structure	Fragment type	Observed *m/z*	Calculated *m/z*	Δ*m/z*
N	B	225.896	226.06859	0.172
NH	B	388.001	388.12141	0.120
N2H	B	550.030	550.17423	0.144
2NH	B	591.839	591.20078	0.639
NHF	B	534.012	534.17932	0.167
2N2H	B	753.076	753.25360	0.177
N2HF	B	696.041	696.23214	0.191
2N2HF	B	898.996	899.31151	0.315

Table S20. List of product ion from *O*-glycan ISD product (*m/z* 940) from PSM with AHB/Na in Fig. S29.

Structure	Fragment type	Observed *m/z*	Calculated *m/z*	Δ*m/z*
N	B	226.004	226.06859	0.065
NH	B	387.961	388.12141	0.160
2N	B	428.976	429.14796	0.172
NHF	B	533.971	534.17932	0.209
2NH	B	590.960	591.20078	0.241
3NH	B	794.039	794.28015	0.241
2NHF	B	737.023	737.25869	0.235
3NHF	B	940.030	940.33806	0.308

Table S21. List of product ion from *O*-glycan ISD product (*m/z* 1102) from PSM with AHB/Na in Fig. 6(a).

**Structure**	**Fragment type**	**Observed *m/z***	**Calculated *m/z***	**Δ*m/z***
NH	B	388.270	388.12141	0.149
NHF	B	534.320	534.17932	0.141
2NH	B	591.415	591.20078	0.214
2N2H	B	753.562	753.25360	0.308
3NH	B	794.634	794.28015	0.353
2N2HF	B	899.730	899.31151	0.419
3N2H	B	956.619	956.33297	0.286
3N2HF	B	1102.559	1102.39088	0.168

Table S22. List of product ion from *O*-glycan ISD product (*m/z* 1120) from PSM with AHB/Na in Fig. 6(b).

**Structure**	**Fragment type**	**Observed *m/z***	**Calculated *m/z***	**Δ*m/z***
NH	B	387.893	388.12141	0.228
NH	C	405.928	406.13197	0.204
NHF	B	534.026	534.17932	0.153
2NH	B	591.043	591.20078	0.158
2NH	C	609.022	609.21134	0.190
2NHF	B	737.118	737.25869	0.140
2N2H	B	753.078	753.25360	0.176
2N2H	C	771.072	771.26416	0.192
3NH	B	794.137	794.28015	0.143
2N2HF	C	917.190	917.32207	0.132
3N2H	C	974.114	974.34353	0.230
3N2HF	C	1120.025	1120.40144	0.377
